# Coaxial Alginate Hydrogels: From Self-Assembled 3D Cellular Constructs to Long-Term Storage

**DOI:** 10.3390/ijms22063096

**Published:** 2021-03-18

**Authors:** Oleksandr Gryshkov, Vitalii Mutsenko, Dmytro Tarusin, Diaa Khayyat, Ortwin Naujok, Ekaterina Riabchenko, Yuliia Nemirovska, Arseny Danilov, Alexander Y. Petrenko, Birgit Glasmacher

**Affiliations:** 1 Institute for Multiphase Processes, Leibniz University Hannover, An der Universität 1, Building 8143, 30823 Garbsen, Germany; mutsenko@imp.uni-hannover.de (V.M.); khayyat@imp.uni-hannover.de (D.K.); glasmacher@imp.uni-hannover.de (B.G.); 2 Lower Saxony Centre for Biomedical Engineering, Implant Research and Development, Stadtfelddamm 34, 30625 Hannover, Germany; 3 Institute for Problems of Cryobiology and Cryomedicine of the National Academy of Sciences of Ukraine, 23 Pereyaslavsky Street, 61015 Kharkiv, Ukraine; tarusindmitriy@gmail.com (D.T.); yulianemyrovska@gmail.com (Y.N.); alexander_petrenko@cryo.org.ua (A.Y.P.); 4 Institute of Clinical Biochemistry, Hannover Medical School, Carl-Neuberg-Strasse 1, 30625 Hannover, Germany; Naujok.Ortwin@mh-hannover.de; 5 Institute for Biomedical Systems, National Research University of Electronic Technology, 124498 Moscow, Russia; caterinco@mail.ru (E.R.); arseny.danilov@gmail.com (A.D.)

**Keywords:** cell encapsulation, coaxial electrospraying, tissue cryopreservation, core-shell capsules, scaffolds, cellular structures, multipotent stromal cells, thermomechanical stress, RAMAN spectroscopy, swelling

## Abstract

Alginate as a versatile naturally occurring biomaterial has found widespread use in the biomedical field due to its unique features such as biocompatibility and biodegradability. The ability of its semipermeable hydrogels to provide a favourable microenvironment for clinically relevant cells made alginate encapsulation a leading technology for immunoisolation, 3D culture, cryopreservation as well as cell and drug delivery. The aim of this work is the evaluation of structural properties and swelling behaviour of the core-shell capsules for the encapsulation of multipotent stromal cells (MSCs), their 3D culture and cryopreservation using slow freezing. The cells were encapsulated in core-shell capsules using coaxial electrospraying, cultured for 35 days and cryopreserved. Cell viability, metabolic activity and cell–cell interactions were analysed. Cryopreservation of MSCs-laden core-shell capsules was performed according to parameters pre-selected on cell-free capsules. The results suggest that core-shell capsules produced from the low viscosity high-G alginate are superior to high-M ones in terms of stability during in vitro culture, as well as to solid beads in terms of promoting formation of viable self-assembled cellular structures and maintenance of MSCs functionality on a long-term basis. The application of 0.3 M sucrose demonstrated a beneficial effect on the integrity of capsules and viability of formed 3D cell assemblies, as compared to 10% dimethyl sulfoxide (DMSO) alone. The proposed workflow from the preparation of core-shell capsules with self-assembled cellular structures to the cryopreservation appears to be a promising strategy for their off-the-shelf availability.

## 1. Introduction

Alginate has been considered as one of the most abundant marine derived naturally occurring biomaterials for the application in cell-based therapies [1], pharmacology [2], tissue regeneration [3], wound healing [4] and others. The cheapest source of alginates is brown algae (*Phaeophyceae*), whereas the alginates of bacterial origin are the most costly. The source of alginate (marine vs. bacterial, place of seaweed collection), the extraction, purification and modification methods determine the content and ratio of (1,4)-linked β-d-mannuronate (M) and its C-5 epimer α-l-guluronate (G) (M/G ratio) and their alternating sequences (MG). Due to their distinguished properties, such as ease of gelling with divalent metal cations (ionic cross-linking) thus forming an “egg-box” structure [5] and 3D environment close to the extracellular matrix of native tissues, high biocompatibility, low immunogenicity in vivo and controlled biodegradability [6,7,8], alginate hydrogels have been widely used in medicine and medicine-related research [3]. This includes delivery vehicles in cancer treatment [9], wound dressing [4], mammalian cell culture in biomedical studies, tissue regeneration with protein and cell delivery, engineering of various tissues/organs [10,11,12] including bladder regeneration [13], bone tissue engineering [14], and a protective carrier for cryopreservation [15].

Since the 1980s, strategies of cell encapsulation in alginate microspheres have been put into practice for the delivery of clinically relevant cells, cell clusters and organoids as well as therapeutics [16]. Cell-laden alginate beads have been used for immunoisolation [17,18,19,20], treatment of diseases [21] and development of artificial organs (such as bioartificial liver (BAL)) [22]. The properties of alginate hydrogels can easily be tailored by the cross-linking type and duration in order to fulfil specific application requirements. Over the last decades, a number of chemical modifications have been elaborated to improve their stability [18], biocompatibility [23], long-term functions of encapsulated cells [21], their controlled differentiation [24,25,26] and targeted cell delivery [27], to name a few. Numerous reviews, highlighting the effect of alginate type and source, cross-linking methods and chemical modifications regarding the performance of alginate beads, have been published so far [12,28,29].

Solid beads can be produced by a variety of methods [30,31,32], among which electrospraying is advantageous in terms of scaling up the process and the possibility for precise control over the size of the solid beads in the micro- and macroscopic range. This method yields in a low diameter variation given by the adjusted process parameters, such as electric field strength (the applied voltage over the spraying distance). For instance, the value of the applied electric field strength of up to 3 kV/cm was shown not to affect the cell viability, metabolic activity and differentiation capacity after encapsulation over a culture period of 7 days [33]. Moreover, there are still debates addressing the effect of the size of the solid beads on their immunogenicity both in vitro and in vivo. Some authors suggest that smaller, micro-sized solid beads with a size below 500 µm are preferred candidates for cell encapsulation and transplantation due to an improved mass transfer [34]. However, supporting the importance of clinical outcome, other authors underline that the application of macroscopic (sizes above 1500 µm) solid beads results in less intense fibrosis and macrophage infiltration in vivo [35].

Alginate core-shell capsules emerged as promising candidates for transplantation due to a high encapsulation efficiency and mimicry of a native tissue environment. In contrast to solid beads, core-shell capsules have not yet been investigated to such an extent for the cell encapsulation. Hereinafter for consistency and clarity reasons, the term ‚solid beads’ refers to monolithic alginate beads having a homogeneous structure whereas ‘core-shell capsules’ designates alginate beads with a liquid core and prepared by the coaxial electrospraying approach.

For the production of core-shell capsules a coaxial nozzle for the co-flow of alginate (outer membrane) and cell suspension (inner core) is necessary. In the coaxial configuration, an alginate membrane shields the encapsulated cells from the external shear stress, providing a more favourable environment where cells could directly interact with each other which is a prerequisite to form 3D structures. Only a few studies have investigated the formation and development of cell aggregates or clusters in solid beads mostly using immortalized cell lines [36]. Interestingly, cells in clusters were seen to be alive, while individual cells appeared dead. The study by Horiguchi and Sakai compared the behaviour of cell aggregates (made of induced pluripotent stem cells (iPSCs)) in solid and hollow beads [37]. Two additional technological steps, either coating with poly-l-lysine (questionable immunoisolation) or treatment with ethylenediaminetetraacetic acid (EDTA) to prepare hollow capsules were needed to prevent cell aggregate leakage out of the alginate beads. To our best knowledge, the formation of 3D cellular structures in alginate capsules, fabricated using a coaxial nozzle per se (not for co-introducing additional coatings), has not yet been reported.

In order to establish cell therapy supply chain for ready to use core-shell capsules with encapsulated cellular structures, their frozen storage and delivery is inevitable. So far, cryopreservation remains the only clinically feasible option enabling long-term storage of native tissues and tissue-engineered constructs (TECs) using either slow freezing [38] or vitrification [39,40] approaches. Although being advantageous in the ice-free preserving of a wide range of biological objects, vitrification often fails to preserve relatively big and complex biological objects due to an increased cryoprotective agent (CPA) toxicity and requires complex handling [41]. Slow freezing is a prevailing strategy used in biobanks worldwide and is based on the application of lower concentrations of cryoprotective agents (CPAs), precise control over the cooling rate and gradual cell dehydration. However, ice formation is expected to be severely damaging in tissues and organs [42].

In this regard, alginate hydrogels possess a range of favourable properties for their cryopreservation: promotion of devitrification [43], viscoelastic properties contributing to less pronounced thermal stresses, a semipermeable membrane protecting the cells from increased osmotic stresses upon CPA addition and removal. Although the cryopreservation of cell-laden solid beads using slow freezing and vitrification has been elaborated by a number of groups [11,44,45,46,47], little if any information is available on the cryopreservation strategies of cell-free and cell-laden core-shell capsules. Zhao and co-workers have been the first to report on the successful vitrification-based cryopreservation of porcine adipose-derived stem cells within core-shell capsules using low concentrations of cell-penetrating CPAs and suppression of ice formation [48]. This microfluidic-based cryopreservation approach in conventional plastic straws is encouraging but lacks of scaling-up ability. In this work relatively small core-shell capsules having an outer and inner diameter of around 600 and 450 µm (total volume of a single capsule as few as 0.17 µL) without any 3D cellular networks were utilised.

For the first time, we evaluated the parameters of coaxial electrospraying to achieve a reproducible production of core-shell capsules, their structural properties and swelling behaviour, analysed viability and metabolic activity of self-assembled 3D cellular structures formed by MSCs within macroscopic core-shell capsules (outer diameter > 3000 µm, volume ≈ 15 µL) for a period of 35 days as well as investigated the cryopreservation strategies for such clinically relevant cell-laden constructs.

## 2. Results

The efficiency of the production of core-shell capsules is mainly determined by the properties of the respective alginate solution, such as density, viscosity and electric conductivity. As expected, the density and viscosity of low viscosity (LV) alginate solutions decreased with increasing temperature, whereas their electric conductivity increased (Figure S1). In addition, the reproducible production of core-shell capsules using the electrospraying approach requires stability of the solution viscosity during the storage. In this regard, a normalized viscosity of 2.0% alginate solution decreased slightly from 100% to 97% from 1 to 21 days of storage in a conventional refrigerator (~4 °C). After around 5 weeks of storage, a drastic decrease in viscosity to 47% as compared to 100% at day 1, was observed. In this work, core-shell capsules were produced from freshly prepared alginate solutions and considered not to use solutions stored at 4 °C longer than 21 days.

The core-shell capsules and solid beads were produced by the electrospraying approach using single or coaxial nozzles, respectively. First, we optimised the process parameters of the electrospraying to analyse the effect of the applied voltage and the ratio of the flow rate of alginate (compared to that of the inner fluid) on the dimensions of the core-shell capsules (outer and core diameters). Afterwards, MSCs were encapsulated into core-shell capsules and solid beads and their viability, cell–cell interactions and metabolic activity were evaluated. In order to optimise the parameters for the cryopreservation of cell-laden core-shell capsules, a parametric study was conducted using cell-free core-shell capsules to evaluate their integrity after thawing. The selected parameters were used for the studies on the cryopreservation of core-shell capsules with self-assembled 3D cellular structures using conventional and modified cryopreservation approaches.

### 2.1. Effect of the Electrospraying Process Parameters on the Size of Core-Shell Capsules

**Figure 1:**
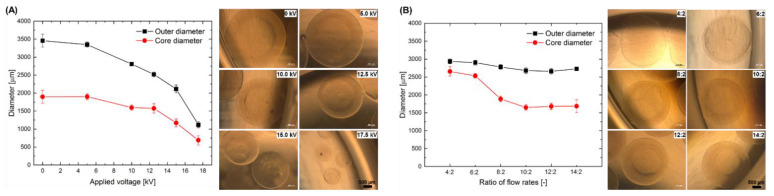
Effect of the applied voltage (**A**) and the ratio of flow rates of alginate to inner fluid (**B**) on the size of the core-shell capsules produced from low viscosity (LV) alginate. Scale bars are 500 µm.

Figure 1A represents the quantitative results on the effect of the applied voltage on the outer and core diameters of the core-shell capsules produced from 1.5% LV alginate using a coaxial nozzle and the electrospraying approach. As expected, both outer and core diameter decrease simultaneously while increasing the applied voltage from 0 to 17.5 kV (electric field strength in the range of 0–2.33 kV/cm). Applying 17.5 kV resulted in the smallest core-shell capsules having an outer diameter of 1116 ± 84 µm. It should be noted that the thickness of an alginate membrane decreased from 797 ± 240 µm to 228 ± 96 µm for 0 and 17.5 kV, respectively. In turn, Figure 1B shows the effect of the increase in the alginate flow rate while keeping the flow rate of the inner fluid (4-(2-hydroxyethyl)-1-piperazineethanesulfonic acid, HEPES) constant, regarding the same capsule parameters. A noticeable decrease in core diameter in this setting can be seen, whereas the outer diameter decreased slightly and remained almost the same for the flow rate ratios above 8:2. The flow rate ratio of 4:2 resulted in core-shell capsules with the thinnest alginate membrane of 158 ± 45 µm. However, under these conditions the electrospraying process was unstable yielding an increased amount of damaged capsules. Therefore, we selected the parameters enabling the most stable electrospraying process for the encapsulation of cells into the core-shell capsules having outer and core diameters around 3000 and 1600 µm, respectively.

### 2.2. Evaluation of Structural Properties of Core-Shell Capsules

**Figure 2:**
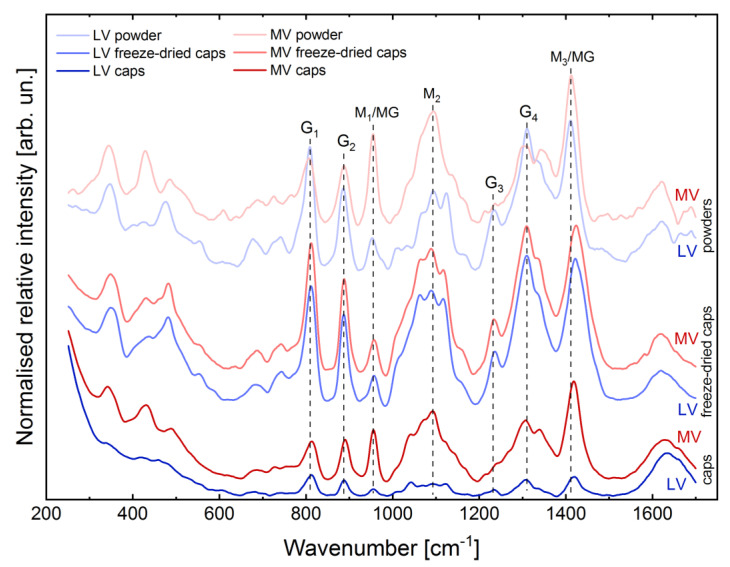
RAMAN spectroscopy of initial materials (powders) as well as produced core-shell capsules before (caps) and after freeze-drying (freeze-dried caps). M_1_–M_3_ and MG are the main bands associated with M- and MG-blocks, respectively; G_1_–G_4_ are the main bands associated with G-blocks; LV and MV are low (high-G) and medium (high-M) viscosity alginates, respectively.

RAMAN spectroscopy was conducted in order to analyse the initial powders of LV and medium viscosity (MV) sodium alginate as well as core-shell capsules produced from these powders before and after freeze-drying. Figure 2 shows the fingerprint region (250–1700 cm^−1^) of the RAMAN spectra of these materials. The region 2800–3700 cm^−1^, containing the band at 2938 cm^−1^ and a broad band with a peak at around 3400 cm^−1^ corresponding to the C-H stretching vibration and O-H stretching vibration of water [49], respectively, is not shown due to no pronounced changes in the intensities and positions of the respective bands, when comparing initial powders and produced core-shell capsules.

In general, the RAMAN spectra of alginates in the fingerprint region can be characterised by three subregions in the wavenumber ranges of 250–700, 700–1250 and 1250–1700 cm^−1^. The presence of the bands in the first two subregions below 1250 cm^−1^ is mainly associated with vibrations of the polymer backbone [50]. In the first subregion, the presence of the main bands at 345, 429, 482 and 675 cm^−1^ (deformation of pyranosyl rings and C–O–C vibration of glycosidic linkage) is observed. The second subregion contains the characteristic bands at 742 cm^−1^ (ring breathing), 808, 887 and 953 cm^−1^ (skeletal C–C, C–O stretching and C–C–H, C–C–O bending modes) as well as quadruple bands at around 1035–1064, 1090–1098, 1124 and 1234 cm^−1^ (C–O symmetric stretching and glycosidic ring breathing). Lastly, the third subregion contains the most intense bands at 1309 and 1334 cm^−1^ (C-H deformation vibration), 1412 (symmetric COO^–^ stretching vibration) and 1621 cm^−1^ (asymmetric COO^–^ stretching vibration) [50]. The RAMAN spectra of LV and MV alginates differ noticeably, suggesting a different content of the respective G- and M-blocks as well as alternating MG-blocks, which results from different intensity ratios of the bands situated at 953 (M_1_/MG), 1090 (M_2_) and 1413 cm^−1^ (M_3_/MG, M- and MG-blocks) and 808 (G_1_), 887 (G_2_), 1232 (G_3_), 1310 cm^−1^ (G_4_, G-blocks).

When comparing the RAMAN spectra of initial powders and capsules before freeze-drying, no noticeable change in the intensity ratios of the bands attributed to G-, M- and MG-block content was observed. Here, the main difference was found in the position of the band at 1412 cm^−1^, which can be associated with the participation of Ca^2+^ ions in cross-linking and thus formation of the “egg-box” structure. In the RAMAN spectra of the capsules, the respective band at 1418 cm^−1^ is shifted by 6 cm^−1^, as compared to initial powders. Taking into account the RAMAN spectra of the freeze-dried materials and comparing them to the RAMAN spectra of the respective initial powders and the produced capsules, one could observe similar intensity ratios of the main bands which determine the M/G ratio. The RAMAN spectra of the MV core-shell capsules are similar to that of freeze-dried LV core-shell capsules as well as LV alginate powder. In the RAMAN spectra of freeze-dried LV and MV core-shell capsules, the band at 1422 cm^−1^ shows a noticeable shift by 10 cm^−1^ towards higher wavenumbers, as compared to its position in the RAMAN spectra of initial powders (1412 cm^−1^); the shift of this band is more pronounced for freeze-dried materials, as compared to the capsules (6 cm^−1^).

### 2.3. Storage Stability and Swelling Behaviour

**Figure 3:**
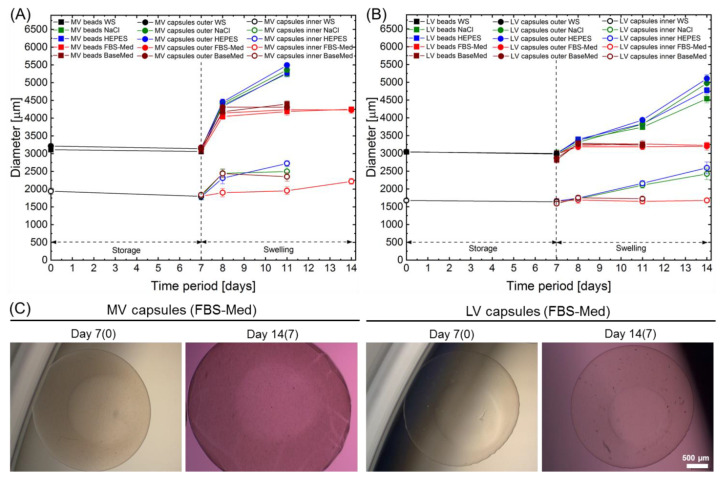
Change in outer (solid beads and core-shell capsules) and inner core (only core-shell capsules) diameters during storage in washing solution (WS) and upon incubation in NaCl and HEPES solutions, fetal bovine serum (FBS)-containing (FBS-Med) and FBS-free (BaseMed) basal medium in the course of further 7 days. (**A**) Solid beads and core-shell capsules produced from 2% medium viscosity (MV) alginate; (**B**) solid beads and core-shell capsules produced from 2% LV alginate. (*n* = 18). Filled squares show solid beads, cycles—core-shell capsules with outer (filled cycles) and inner core diameters (open cycles), different colours correspond to different solutions (black—WS, green—NaCl, blue—HEPES, red—FBS-containing medium, brown—FBS-free medium); (**C**) Bright-field photographs of MV and LV core-shell capsules on day 7 and 14 (days 0 and 7 of swelling) in cell culture medium. Scale bars are 500 µm.

Stability of the core-shell capsules and solid beads, prepared from the LV and MV sodium alginates, was evaluated while storing them in washing solution (WS), containing 20 mM CaCl_2_ for 7 days at 4 °C, followed by the incubation in saline (0.9% NaCl) and 10 mM HEPES solutions as well as fetal bovine serum (FBS) free basal and *cj*aMSCs culture medium in the course of further 7 days to investigate swelling behaviour. Figure 3 represents the results on analysing the changes in diameters of the core-shell capsules and solid beads. As can be seen, storage of cell-free capsules and beads in WS in a refrigerator did not result in noticeable change in the diameters (Figure 3, first 7 days).

The most prominent swelling behaviour (Figure 3, second period of 7 days) is observed for solid beads produced from 2% MV alginate on day 1, where the outer diameter increased by 43%, 41% and 32% (for the solid beads incubated in NaCl, HEPES and cell culture medium, respectively), as compared to MV solid beads before swelling (day 0). The outer and inner core diameters of the core-shell capsules produced from 2% MV alginate showed a slightly lower increase in diameter by 41% (outer shell) and 36% (inner core), 42% (outer shell) and 29% (inner core) as well as 32% (outer shell) and 5% (inner core) for NaCl, HEPES and cell culture medium on day 1, respectively.

The core-shell capsules and solid beads produced from 2% LV alginate exhibited a significantly lower swelling rate on day 1, as compared to the solid beads produced from 2% MV alginate (*p* < 0.0001). On day 1, the outer diameter of LV solid beads increased by 12%, 14% and 8% (for the solid beads incubated in NaCl, HEPES and cell culture medium, respectively), as compared to LV solid beads before swelling (day 0). In turn, the outer and inner core diameters of the core-shell capsules produced from 2% LV sodium alginate increased by 12% (outer shell) and 7% (inner core), 12% (outer shell) and 5% (inner core) as well as 7% (outer shell) and 2% (inner core) for NaCl, HEPES and cell culture medium on day 1, respectively. Interestingly, comparing the type of the solution used for the swelling incubation of the capsules and the beads within the FBS-containing medium resulted in a less prominent increase in the diameters for both LV and MV alginates, as compared to NaCl and HEPES solutions (*p* < 0.0001). On the latter days of the swelling experiment (day 4 and day 7), the diameters of the LV core-shell capsules and solid beads increased further, whereas the ones produced from the MV alginate and incubated in NaCl and HEPES dissolved completely on day 7. Comparing the swelling behaviour of the LV and MV core-shell capsules and solid beads, a less pronounced increase in diameter was detected for the capsules and the beads produced from LV alginate. Notably, the incubation of the capsules and beads in the FBS-free and FBS-containing medium did not result in a further increase in the diameters on day 4 and day 7, as compared to day 1. As can be seen from the bright-filed images of the LV and MV core-shell capsules on day 4 and 7 of incubation in the FBS-containing medium, certain deposition on the surface of the MV core-shell capsules is observed, which was not detected before addition of solutions (Figure 3C, day 14(7) vs. day 7(0)).

The results on analysing the water uptake of the core-shell capsules and solid beads presented in Table 1, revealed a higher water uptake for the core-shell capsules (LV—96.4 ± 0.2%, MV—95.9 ± 0.2%) as compared to solid beads (LV—95.8 ± 0.4%, MV—95.1 ± 0.1%). The water content calculated according to the Equation (3) was much higher for the core-shell capsules, as compared to solid beads.

### 2.4. Effect of the Core-Shell Capsules and Solid Beads on the Cell Viability

In order to evaluate the effect of the encapsulation method (core-shell capsules vs. solid beads) on the cell functionality in vitro, two different cell types were used: amnion MSCs derived from the placenta of a common marmoset *Callithrix jacchus* (*cj*aMSCs, viability in long-term culture and cryopreservation) and human dermal MSCs (hdMSCs, metabolic activity).

**Figure 4:**
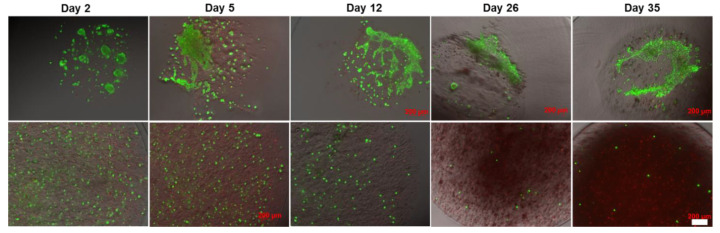
Representative fluorescent images of *cj*aMSCs encapsulated in core-shell capsules (**upper row**) and solid beads (**lower row**) for a total period of 35 days in culture. Scale bars are 200 µm.

Figure 4 represents fluorescence microscopy pictures of cells cultured within core-shell capsules (upper row) and solid beads (lower row) for a total period of 35 days stained with Calcein AM and Ethidium Homodimer dyes. During the initial culture period (days 2–3), the viability of cells encapsulated within core-shell capsules and solid beads remained high, with slightly more dead cells visible in solid beads. Notably, the cells within core-shell capsules started to form cellular clusters and inter-cellular networks from day 2. Upon further culture, these cellular constructs remained highly viable with minor presence of dead cells. In contrast to core-shell capsules, the viability of cells encapsulated within solid beads progressively declined and the cells remained isolated over the long-term cultivation period. After 1 week of culture, visibly less than a half of the cells remained alive. At the end of the cell culture on the day 35, only a few viable cells could be detected within the solid beads. Encapsulation of cells in core-shell capsules allowed to maintain a high cell viability and supports the cells in developing cell–cell contacts and 3D assemblies.

**Figure 5:**
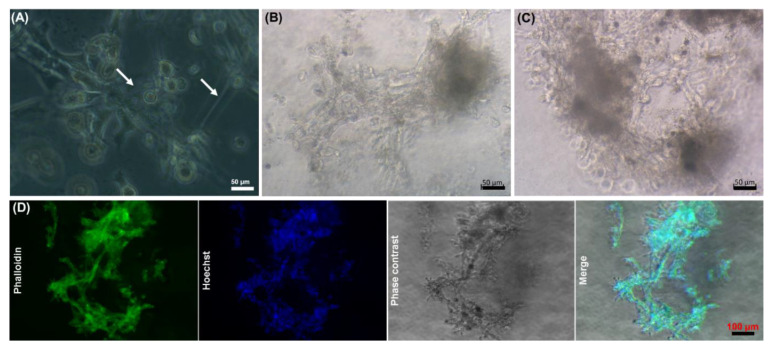
Formation of cellular structures within core-shell capsules in the course of long-term in vitro culture. Bright-field images of cellular formations on day 3 (**A**), day 17 (**B**) and day 35 (**C**). (**D**) Appearance of actin filaments (green fluorescence) and nuclei (blue fluorescence) evaluated using Phalloidin–Hoechst staining on day 17. White arrows in (**A**) show intercellular bridging and cell spreading. Scale bars are 50 µm (**A**–**C**) and 100 µm (**D**).

Establishment of intercellular bridges during in vitro culture is representatively shown in Figure 5. As can be seen, the cells within the core-shell capsules start to form bridges on day 3 (Figure 5A, white arrows) and develop clearly visible interconnected cellular structures on day 17 (Figure 5B) and day 35 (Figure 5C). Typical images of actin cytoskeleton integrity (Phalloidin-Hoechst fluorescence staining) in such cellular assemblies is shown in Figure 5D.

**Figure 6:**
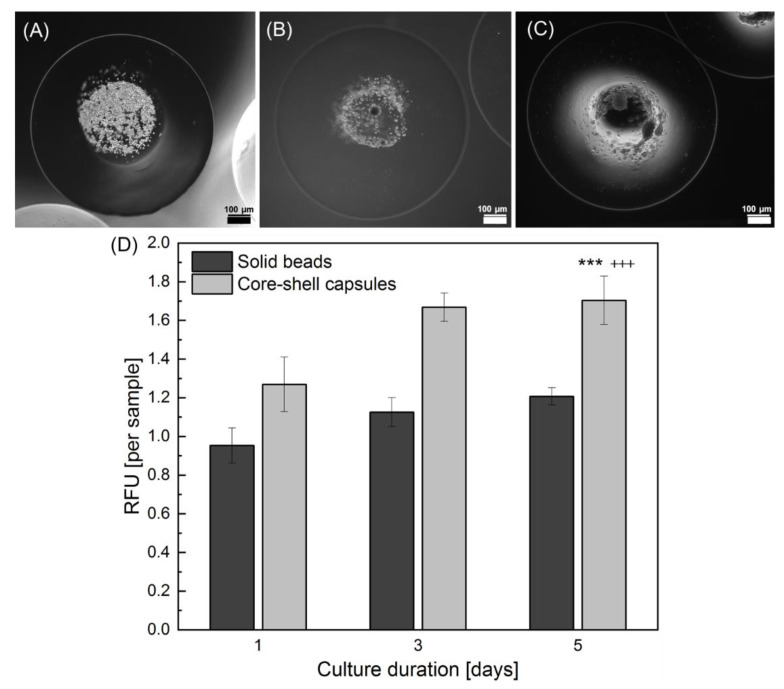
Human dermal MSCs encapsulated in core-shell capsules and solid beads. HdMSCs within core-shell capsules after encapsulation (**A**) and on day 3 (**B**) and 5 (**C**) in culture. (**D**) Metabolic activity of hdMSCs encapsulated in core-shell capsules and solid beads during culture for 5 days assessed by Alamar Blue test. Statistical analysis was performed using one-way Analysis of Variance (ANOVA) (*p* = 0.05) and a Tukey’s multiple comparison test. The data are presented as a mean and standard deviation. In (**D**) *** data are significantly different (*p* < 0.001) compared to solid beads on days 1, 3 and 5, +++ data are significantly different (*p* < 0.001) compared to core-shell capsules on 1st and 3rd day of culture. The outer diameter of the capsules is about 1000 µm, whereas the core diameter is approximately 420 µm. Scale bars are 100 µm.

Application of the encapsulation method via a coaxial electrospray was also analysed for hdMSCs. Specifically the cell metabolic activity within core-shell capsules and solid beads was analysed using Alamar Blue test for 5 days in culture conditions. Figure 6 represents microscopic images of the core-shell capsules directly after hdMSCs encapsulation (A) and following subsequent culture for 3 (B) and 5 (C) days.

As can be seen, the cells start to form aggregates on day 3 post-encapsulation resulting in a formation of more complex cellular structures at day 5. The results on metabolic activity of hdMSCs encapsulated in core-shell capsules and solid beads is shown in Figure 6D. At all tested time intervals after encapsulation, the metabolic activity within the core-shell capsules was not only significantly higher as compared to cells within solid beads but also demonstrated a time-dependent increase. In particular, the encapsulation of cells in core-shell capsules yielded in significantly higher values of metabolic activity on day 5 as compared to that on day 1 and day 3 after encapsulation (*p* < 0.001). Moreover, the metabolic activity of hdMSCs within core-shell capsules on day 5 was significantly higher (*p* < 0.001) as compared to that of hdMSCs within the solid beads on the same day after encapsulation.

**Figure 7:**
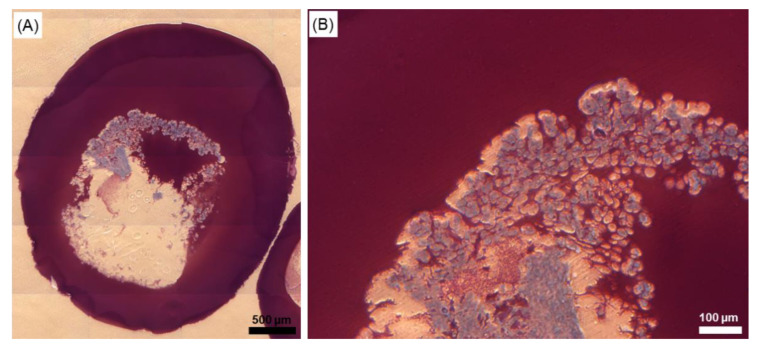
Toluidine blue staining profile of cellular networks within core-shell capsules after 35 days of culture. Picture (**B**) represents a higher magnification of (**A**). Cellular structures are shown in blue whereas the shell of an exemplary core-shell capsule is stained in red-purple. Scale bars are 500 µm (**A**) and 100 µm (**B**).

To analyse the internal appearance of core-shell capsules laden with cellular structures, 5 µm histological sections were prepared and stained with toluidine blue (Figure 7). As can be observed, the sample has a hollow core and a solid outer shell and is stained in different gradations of red-purple colour whereas cellular structures appear blue, contrasting spread-out cellular networks.

### 2.5. Selection of Parameters for the Cryopreservation of Cell-Free Core-Shell Capsules

**Figure 8:**
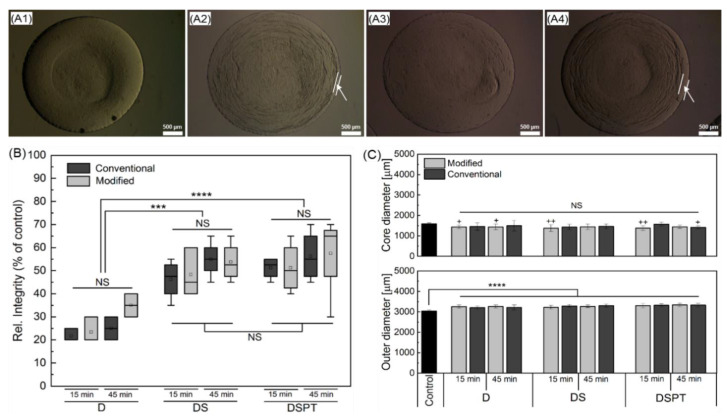
The effect of the cryopreservation on the integrity and size change of cell-free core-shell capsules after thawing. (**A**) Representative bright-field images of core-shell capsules before (**A1**) and after freezing under protection of 10% DMSO ((**A2**), D), 10% DMSO and 0.3 M sucrose without ((**A3**), DS) and with capsule pretreatment with 0.1 M sucrose 24 h before freezing ((**A4**), DSPT) using the modified “in air” approach. White arrows on (**A2**,**A4**) indicate a possible effect of the CPA type on the preservation of the membrane integrity. Scale bars are 500 µm. (**B**) Relative capsule integrity according to the gradation of damage as detailed in Section 4.4.1. Kruskal–Wallis-test followed by post hoc Dunn’s multiple comparison (*p* < 0.05): boxes show 25–75% data range, line and open quadrat markers in the box—median and mean, respectively, and whiskers—1.5IQR; NS—not significant, *** *p* < 0.001, **** *p* < 0.0001 (*n* ≥ 3). Statistics indicates significant improvements in the relative capsule integrity after cryopreservation using 0.3 M sucrose to 10% DMSO as compared to 10% DMSO alone. (**C**) The effect of cryopreservation on the outer and core diameter after thawing as compared to core-shell capsules before freezing (control). One-way ANOVA (*p* = 0.05) (normal distribution) with a Tukey’s post-hoc multiple comparison test. The data are presented as mean and standard deviation. In (**C**): **** the data are significantly different (*p* < 0.0001) compared to the control, + and ++ define that the data are significantly different (*p* < 0.05, *p* < 0.01, respectively) as compared to the control (*n* ≥ 12).

First cryopreservation studies were performed on cell-free core-shell capsules utilising the pre-selected parameters for the freezing of cell-laden core-shell capsules. Figure 8 shows the qualitative and quantitative results on the alterations in shape and size of cell-free core-shell capsules after freezing and thawing using conventional and modified procedures. Representative bright-field images of core-shell capsules (upper panel) and quantification of the capsule integrity (lower panel) after thawing (normalized to the total capsule integrity before freezing taken as 100%) are presented. Figure 8A1–A4 illustrates non-frozen samples and samples cryopreserved using 10% DMSO (D), 10% DMSO with 0.3 M sucrose including without (DS) and with capsule pretreatment (DSPT) with 0.1 M sucrose 24 h before freezing, respectively.

Bright field images of cell-free core-shell capsules before and after cryopreservation (Figure 8A) suggest that the application of sucrose is advantageous in terms of preservation of the structure of the alginate membrane (white arrows in Figure 8A2,A4). Of note, the application of sucrose resulted in a preservation of the alginate membrane at a higher extent, as indicated by whiter arrows. Detailed overview of bright-field images of core-shell capsules after thawing can be consulted in Figure S2.

As can be seen, the addition of 0.3 M sucrose to the freezing medium significantly improves the capsule integrity after thawing for not pretreated (*p* < 0.001) and pretreated (*p* < 0.0001) samples, as compared to DMSO-frozen ones. Application of an additional pretreatment step for 24 h before freezing (DSPT) did not show a significant improvement in post-thaw capsule integrity (*p* > 0.05) as compared to the DS group. In turn, the variation of CPA loading time before the cryopreservation (15 min vs. 45 min) and the method of cryopreservation (modified vs. conventional) did not result in a significant increase of post-thaw integrity of the core-shell capsules (*p* > 0.05).

Comparing the change in size of core-shell capsules before and after cryopreservation, a total increase in the outer diameter and decrease in the core diameter by around 8% was detected (Figure 8C). Within the tested cryopreservation parameters, the values for the outer diameter were significantly different compared to that of the unfrozen core-shell capsules (*p* < 0.0001). In addition, no significant difference in core diameter was observed among the cryopreservation groups (D vs. DS and DSPT) and strategies (conventional vs. modified). However, a core diameter was significantly decreased (*p* < 0.05) for the parameters marked by asterisks in Figure 8C as compared to core-shell capsules before cryopreservation. The observed alterations in the outer and core diameters could be associated with a partial increase in the membrane thickness of the core-shell capsules after thawing.

### 2.6. Effect of the Cryopreservation on Cell-Encapsulated Core-Shell Capsules

For the validation of cryopreservation of cell-laden core-shell capsules, a CPA cocktail containing 10% DMSO and 0.3 M sucrose with the pretreatment step was selected, based on the above presented results. The latter step was dictated by the need to provide intracellular cryoprotection of the cells and thus the beneficial effect of sucrose-pretreatment as we have previously shown [51,52].

**Figure 9:**
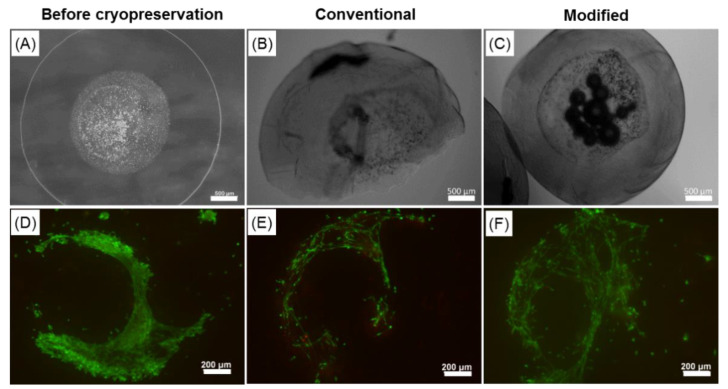
Bright field (**A**–**C**) and fluorescent images (**D**–**F**) of *cj*aMSCs encapsulated in core-shell capsules before freezing and after thawing using conventional and modified approaches. Conventional cryopreservation was associated with a noticeable damage to the capsules’ integrity (**B**) and decrease in cell viability (**E**). The cell viability was analysed 24 h after thawing using live-dead assay indicating live cells in green fluorescence and dead cells—in red. A much higher amount of viable cells within cellular networks is seen in samples protected with the modified protocol (**F**). Scale bars are 500 µm (**A**–**C**) and 200 µm (**D**–**F**).

Conventional and modified cryopreservation approaches were compared with respect to preserving delicate 3D cellular structures formed within core-shell capsules. Figure 9 represents comparative results on the integrity of core-shell capsules as well as the viability of enclosed *cj*aMSCs before (Figure 9A,D) and after cryopreservation using conventional (Figure 9B,E) and modified approaches with 10% DMSO and 0.3 M sucrose with a sucrose-pretreatment step (Figure 9C,F). Using conventional cryopreservation, considerable mechanical damage to core-shell capsules took place resulting in the complete disintegration of the capsule form, membrane damage and accompanied release of the encapsulated cells (Figure 9B). Moreover, conventional cryopreservation resulted in a decreased post-thaw cell viability as determined by live-dead staining (Figure 9E). In contrast, modified cryopreservation which implies removal of CPAs excess after loading and fast addition of pre-warmed cell culture medium upon thawing, yielded in a better preservation of the capsule integrity (Figure 9C) and a noticeably higher cell viability after thawing (Figure 9F).

## 3. Discussion

Among a considerable variety of marine-derived biopolymers alginate presents an immense industrial importance (food, pharmaceutical, printing, cosmetic and biomedical industries as well as wastewater treatment) [53]. As a main structural component of marine brown algae, constituting up to 40% of their dry matter, alginate can be obtained from farmed algae (primarily the genera *Laminaria* and *Macrocystis*) at a relatively low cost (it can also be produced by some bacteria) [54].

Alginate encapsulation of clinically relevant cells has been considered as an important step towards efficient cell-based therapies, immunoisolation and tissue regeneration. Porous and semipermeable alginate membrane allows the diffusion of nutrients to the encapsulated cells and a controlled release of therapeutics to the implantation site [55,56]. Moreover, a mild environment within alginate hydrogels, ease of tuning their mechanical properties as well as mimicking the extracellular matrix is of special importance for survival, long-term functionality and controlled differentiation of encapsulated stem cells. In order to create frozen stocks of such prospective materials with regenerative potential, their long-term preservation has to be considered while ensuring hydrogel integrity and vital cellular characteristics after storage. In this paper, we demonstrated the formation of self-organized 3D-like cellular structures within core-shell capsules made from low viscosity alginate using the electrospraying method. Moreover, we proposed a strategy for the cryopreservation of cell-laden core-shell capsules to protect the capsule integrity and cell viability at a relatively high level. To our knowledge, this is the first study reporting on both the formation of self-organized 3D cellular structures within core-shell capsules as well as steps towards their cryopreservation.

### 3.1. Reproducible Production of Core-Shell Capsules Using Coaxial Electrospraying

The production of core-shell capsules can be performed by a number of methods including air-flow, electrospraying and microfluidics. The common idea behind them is a coaxial flow of two liquids: alginate as an outer membrane and a cell suspension as a core (for microfluidics—different Y- or T-junctions). Due to the high variability in the setup configurations a direct comparison between the effects of the process parameters on the specific properties of solid beads reported in literature is rather complicated and misleading. Generally, while increasing an electric field during the electrospraying approach, the size of the solid beads decreases. We [30] and others [57] have comprehensively demonstrated different regions in the dependency between the applied electric field strength and the size of formed solid beads. Our current results for core-shell capsules follow the same trend. In case of core-shell capsules, the thickness of the alginate membrane can be varied in the following manner: increasing the flow rate of the outer alginate solution while keeping the flow rate of the inner one constant results in a thicker alginate membrane of the core-shell capsules (Figure 1B). Interestingly, Figure 1B contains several regions: till 6 mL/h, 6 to 8 mL/h and from 8 mL/h. This could be attributed to the effect of the electrical conductivities of the utilised solutions (Figure S1). We hypothesise that the first region is mainly determined by the inner fluid (HEPES), having a lower electrical conductivity at room temperature, which could result in an increased core diameter. While increasing the flow rate of the alginate solution having higher electrical conductivity to 6 mL/h, we achieve a transition (metastable) region, where the outer and core diameters decrease. The third region with a flow rate of the alginate solution from 8 mL/h is determined by the higher amount of alginate solution during the formation of droplets and thus, as it is the case for the effect of voltage, results in slightly smaller capsules and decreased core diameters. An increase in the alginate concentration from 1.5% to 2.0% had no noticeable impact on the outer diameter, whereas the inner core diameter was smaller for the higher alginate concentration. This property could be useful for designing the most optimal configurations of core-shell capsules for different applications: core-shell capsules produced from lower alginate concentrations are less rigid, have a thinner alginate membrane and could be regarded as advantageous for transplantation. On the other hand, core-shell capsules fabricated from a higher alginate concentration are more stable to shear stress; they could be more appropriate for the dynamic cultivation of encapsulated cells in a bioreactor, for instance, for iPSCs expansion and development of efficient BAL approaches.

For the application of cell-laden core-shell capsules in clinics, high numbers have to be produced, which is reflected by the efficiency of process scaling up. In our case, an increase in the flow rates of the alginate and HEPES solutions from 8:2 to 16:4 (mL/h:mL/h), while keeping their ratio (4:1) constant did not give rise to increased outer and core diameters (Figure S3). This suggests that the coaxial electrospraying process can be scaled up to potentially produce a required number of core-shell capsules for clinical applications.

### 3.2. Structural Peculiarities and Swelling Behaviour of the Core-Shell Capsules and Solid Beads

In order to shed light on the structural properties of core-shell capsules in wet and dried states as well as to understand the effect of cross-linking on a molecular level, RAMAN spectroscopy has been conducted. The RAMAN spectra of the produced capsules were compared to those in dried state and to initial alginate powders. The results indicate that the powder of LV alginate has a higher G-block content (lower M/G ratio), as compared to the MV alginate powder (higher M/G ratio) (Figure 2). This results from a higher ratio of the intensities of the RAMAN bands at 805/887 cm^−1^ (G-blocks) vs. 1090–1098/1412 cm^−1^ (M- and MG-blocks). As revealed by Salomonsen et al. [58] as well as Pielesz and Bak [59], the content of G- and MG blocks and thus the M/G ratio can be analysed, based on the variations in the intensity ratios of the bands as follows: the M/G ratio increases if the intensities of the peaks at 708 (not present in our RAMAN spectra of alginates), 953, 1090 and 1413 cm^−1^ increase, whereas it decreases with an increase in the intensities of the bands at 808, 887, 1232, 1310 cm^−1^. Our RAMAN results suggest different contents of residues (and thus M/G ratio) in the studied alginates, which is in agreement with the literature [60,61]. It should be pointed out that in the respective datasheets of the alginates used in this study limited information from a manufacture regarding the M/G-ratio (content of G- and M-blocks) was available.

Upon cross-linking, one calcium ion binds two COO^−^ groups in the alginate structure thus forming the so called “egg-box” structure [62]. It is noteworthy that mainly G-blocks participate in the cross-linking with calcium, although some information stating that alternating MG-blocks exhibit additional binding to calcium is available [8,63]. After cross-linking a shift of the band situated at 1412 cm^−1^ (symmetric stretching of the COO^−^ group) by 6 cm^−1^ towards a higher wavenumber compared with initial powders can be observed; it becomes more pronounced in the RAMAN spectra of the freeze-dried materials (shift by 10 cm^−1^) and indicates an interaction of the alginate with calcium [50]. In general, the shift of this peak is indicative of the content of metal cations in the alginate gel [64]. Interestingly, the RAMAN spectra of both freeze-dried LV and MV core-shell capsules are similar to that of LV sodium alginate powder, which has a higher content of G-blocks (lower M/G ratio). The large shift of the band at 1412 cm^−1^ in the RAMAN spectra of freeze-dried alginates could be attributed to a more distinct separation of the molecular vibration, where the intensities of the peaks associated with the content of G-blocks in the alginate structure become higher and thus dominate over the RAMAN spectra of MV alginate after freeze-drying. Moreover, a shift of the band at 1098 cm^−1^ (glycosidic ring breathing mode) by 10 cm^−1^ towards lower wavenumbers could serve as an indirect consequence of the cross-linking, resulted from the weakening of C–C and C–O bonds due to binding of calcium to alginate [50]. Although RAMAN spectroscopy is an excellent tool to investigate the structural properties of biomaterials, a far distinct separation between G-, M- and MG-blocks in the final capsules and initial materials could become possible with the application of surface-enhanced RAMAN scattering (SERS), as revealed by Campos-Vallette et. al., suggesting an advantage of this technique in analysing block content in alginates [65]. In order to get insights into the absolute content of G-, M- and MG blocks in the utilised alginate, certain standards of alginates with a dominating content of the respective residues have to be used as well as a more precise analysis of RAMAN spectra (e.g., principle component analysis, PCA) has to be conducted.

Storage stability, swelling behaviour and viscoelastic properties of core-shell capsules and solid beads are closely linked to the M/G ratio in the alginates. We analysed the stability and swelling behaviour by measuring the diameters during the incubation in a washing solution (storage in a refrigerator) for 7 days followed by further incubation in 0.9% NaCl, 10mM HEPES, FBS-free and FBS-containing medium for 7 days (swelling) (Figure 3). The reason behind the selection of 0.9% NaCl was that it is commonly used for transplantation, whereas HEPES is a buffer solution known to control the pH in the range of 6.8 to 8.2 and, in fact, was also a base for the preparation of all the solutions used for the production of core-shell capsules and solid beads in this study. Our results suggest that the storage of the core-shell capsules and solid beads in WS for 7 days at 4 °C does not have noticeable effects on the diameter. The swelling results indicate that the core-shell capsules and solid beads produced from MV alginate (high-M alginate as revealed by RAMAN) swell at significantly higher rates in NaCl, HEPES, FBS-free and FBS-containing medium, as compared to the ones produced from the LV alginate (high-G alginate based on RAMAN). This could be attributed to the lower concentration of calcium in the hydrogels produced from the high-M MV alginate, as compared to the ones produced from high-G LV alginate. In turn, sodium ions may contribute to the swelling by replacing calcium ions thus affecting stability of the MV capsules and beads over time to much higher extent. Interestingly, the core-shell capsules and solid beads produced from both LV and MV alginate exhibited lower swelling rates in the basic and FBS-containing medium. A less pronounced swelling during long-term in vitro culture could predominantly be attributed to the presence of divalent metal cations (Ca^2+^ and Mg^2+^) in the culture medium (their total salt concentration is around 3 mM). In support of our results, Ramos et al. revealed that the hydrogels produced from the alginate having a lower M/G ratio and a lower molecular weight (similar to LV alginate in our study) have been found to be more stable for the cultivation of probiotic bacteria [66]. A number of studies have analysed the swelling behaviour of the solid beads produced from different types of alginates (high-G or high-M) cross-linked with a range of divalent metal cations, such as Ca^2+^ and Ba^2+^. Mørch et al. revealed that Ca^2+^ binds to G- and MG-blocks, whereas Ba^2+^ participates in the cross-linking by binding to G- and M-blocks [7]. The authors found out that the solid beads produced from the high-G alginate and cross-linked with 50 mM CaCl_2_ tended to swell to a much greater extent in 0.9% NaCl solution as compared to the beads produced from high-M alginate, which in part contradicts to our results. It was also revealed that addition of as low as 1 mM BaCl_2_ into cross-linking solution noticeably reduced extensive swelling of solid beads due to higher affinity of barium cations, as compared to calcium, in interacting with alginate carboxyl groups. Along with the fact that the authors did not present statistical analysis, inappropriate handling could not be excluded, since only the solid beads produced from high-G alginate and cross-linked with calcium did not follow the general swelling trend, as compared to other alginate types and cross-linking agents. In support of our results, other authors [67] suggest a higher affinity of calcium cations to high-G alginates (such as from *L. digitata*) as compared to high-M alginates (such as *L. hyperborean stipes*) and thus a higher stability of Ca-gelled high-G alginate beads in physiological solutions. In our case, the osmolality of the solutions increased slightly in the following order: NaCl (288 ± 4 mOsmol/kg), HEPES (303 ± 1 mOsmol/kg) and basal vs. complete cell culture medium (333 ± 0 vs. 334 ± 1 mOsmol/kg).

### 3.3. Core-Shell Capsules Are Superior to Solid Beads in Terms of Long-Term Cell Culture and Formation of 3D Self-Assembled Cellular Structures

**Figure 10:**
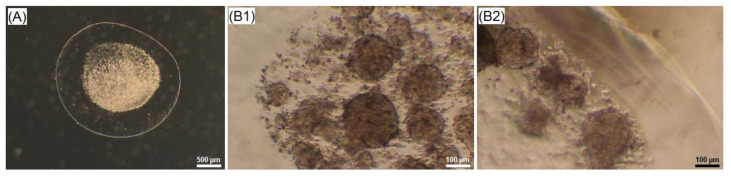
Bright-field images of hESCs within core-shell capsules directly after encapsulation (**A**) and after 4 days of cultivation in StemMACS iPS-Brew XF (**B1**,**B2**). The cells were encapsulated at a concentration of 5 × 10^6^ cells/mL. Scale bars are 500 µm (**A**) and 100 µm (**B**).

The formation of 3D cellular structures is another important point to consider. Unmodified alginates do not support the attachment of mammalian cells due to the absence of cell attachment ligands. Therefore, in most cases the cells encapsulated within solid beads do not form tight cell–cell contacts. For this reason, a number of alginate modifications with peptides, including the sequence arginine-glycine-aspartic acid (RGD) [68] and integrin binding ligands [69], were introduced to improve the cell attachment and growth. While hepatocyte and iPSCs were able to grow and form clusters within unmodified solid beads [70,71], little is known about the formation of self-assembled 3D-like cellular structures by stem cells presumably due to the cell migration, communication and association within the core-shell capsules. In this regard, the encapsulation of embryonic and pluripotent stem cells into beads containing a hollow core was reported to be advantageous, as compared to solid beads in terms of cell cultivation and formation of aggregates [37]. In hollow capsules, aggregates remained encapsulated on day 10, whereas their release from the solid beads was observed on day 4 after encapsulation. In this context, our preliminary results on the encapsulation of human embryonic stem cells (hESCs) into core-shell capsules suggest the efficiency of the coaxial encapsulation method, confirmed by the formation of viable cellular aggregates with a diameter of around 100 µm on day 4 of culture (Figure 10). After encapsulation, the cells also expressed stage-specific embryonic antigen-4 (SSEA4) (data not shown).

Here we have indicated that another cell type specifically hdMSCs also formed 3D cellular structures during culture within core-shell capsules. An increased level of cell metabolic activity assessed by Alamar Blue test on day 5 of culture may obviously be explained by the growth and proliferation of hdMSCs in the core-shell capsules in contrast to solid beads. Interestingly, our preliminary results on the encapsulation of *cj*aMSCs resulted in cell aggregation within the core-shell capsules produced from the 2% MV alginate solution. In this case, the viability of the cells within these aggregates decreased over the cultivation period (Figure S4). Applying LV alginate for the cell entrapment within core-shell capsules resulted in the unexpected formation of self-organised cellular constructs, as revealed by our long-term cultivation study and analysis of cell viability. Interestingly, over the long cultivation period the cells formed cell–cell bridges and started to spread colonising a core. The formed structures remained viable over at least 35 days of culture. It could be speculated that while some cells die their components (e.g., proteins) could remain in a core and adsorb to an inner membrane. This could contribute to the modification of the membrane allowing cell attachment and spreading. In our previous studies we revealed that *cj*aMSCs encapsulated in solid beads were able to differentiate into adipogenic and osteogenic lineages after encapsulation [33,72]. The same tendency we also expect for core-shell capsules which remains to be investigated. How the cell functionality on more comprehensive cellular and molecular levels will be realised within core-shell capsules and in the framework of 3D cellular structures including after cryopreservation remains to be investigated.

### 3.4. Steps towards Efficient Cryopreservation

The development of cryopreservation methods for the efficient protection of the structural integrity and viscoelastic properties of alginate hydrogels for the long-term storage is among the essential factors determining their commercial success in cell-based therapies and clinical outcome. In the case of a slow freezing approach, the integral effect of the freezing, storage and thawing processes is of upmost importance, not only for the entire stability of the core-shell capsules but also the post-thaw functional properties of encapsulated cells and self-organized 3D cellular constructs. The effect of the slow freezing process on the integrity of solid beads has recently been investigated by our group [73]. Using cryomicroscopy, we reported a correlation between the integrity of cell-free solid beads (mean diameter 300 µm) produced from 1.5% MV alginate and the temperature of spontaneous ice formation. In particular, ice nucleation at higher temperatures was shown to be detrimental to the overall structure of solid beads compared to lower temperatures; however, no visible contribution of either the DMSO concentration in the range of 5–10% or its loading time (15/45 min) to bead integrity was observed. It is known that the extent of supercooling correlates to ice crystal size in an inverse manner. Therefore, larger ice crystals forming at higher nucleation temperatures are assumed to be more damaging to the structure of alginate beads (although the opposite effect is expected for a cellular component). In the current study, cryomicroscopic analysis has not been performed due to the large dimensions of the prepared core-shell capsules. In this context, the infrared thermography that provides non-invasive monitoring of multiple freezing/thawing events may be very informative and instrumental for the high-throughput analysis of core-shell capsules during cryopreservation [74,75].

**Figure 11:**
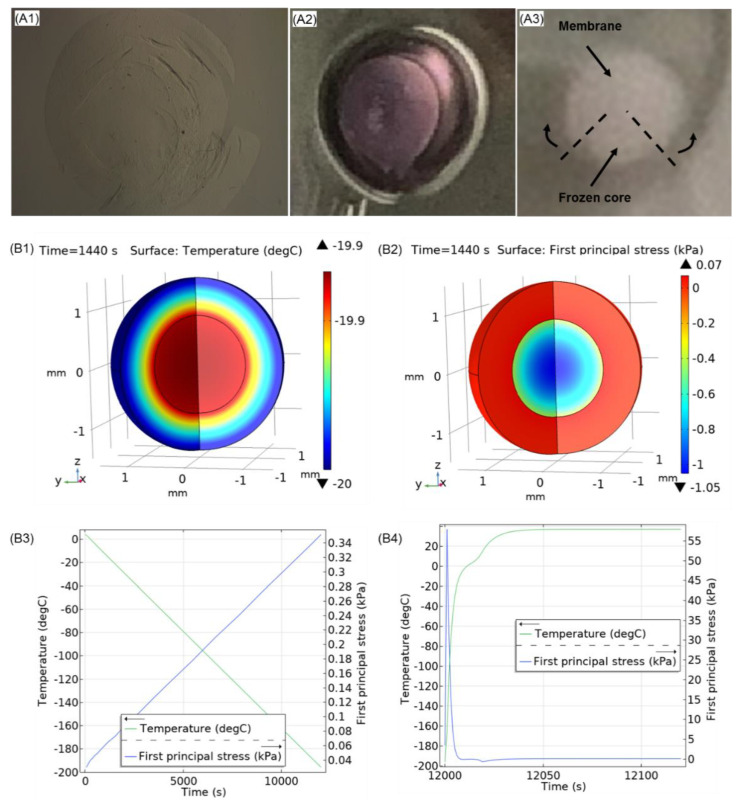
Representative images of cell-free core-shell capsules highlighting the shell damage (**A**) and modelling results for the temperature as well as the maximum principal stress distributions during freezing and thawing (**B**). (**A1**,**A2**) Show a bright-field picture and photo of a damaged core-shell capsule after thawing, respectively, whereas (**A3**) is an enlarged view of a damaged core-shell capsule indicating potential places with increased thermal stresses (curved arrows). The dashed lines define release of a frozen core from a capsule. The core-shell capsules were frozen in a 12-well plate with the application of 10% DMSO and 30 min loading time. Cryopreservation was performed in an Asymptote VIA Freeze Research (Cambridge, United Kingdom) controlled-rate freezer with a cooling rate of 1 K/min down to −70 °C. Warming was conducted passively “in air” without the application of a pre-warmed water bath. (**B1**) Temperature distribution and (**B2**) spatial principal stress distribution at −20 °C during slow freezing of a model core-shell capsule in a cryovial; temperature and principal stress dependencies during cooling (**B3**) and warming (**B4**).

The main challenge in slow-freezing cryopreservation of hydrogel macrospheres with encapsulated cellular constructs is associated with their high water content. Typically, solid beads contain around 95–99% water and have a homogeneous structure [76]. In contrast, core-shell capsules have much more heterogeneous structure and their disintegration due to more pronounced differential thermal expansion at the interfaces liquid core—solid shell upon phase transitions could occur. These freezing damages are anticipated to be followed by the release of entrapped cells and the reduction of their functional properties. When comparing the core-shell capsules and solid beads produced from LV and MV alginates, we found that core-shell capsules have higher water content as compared to solid beads, which is in accordance with the available information in literature [76]. This could be associated with the different distribution of hydrogel density in a monolythic solid bead vs. a core-shell capsule containing bulk liquid in a core. In this regard, the outer membrane-core interface, where increased mechanical stresses unfold is the critical determinant for the maintenance of the integrity of the whole construct during freezing and thawing. It is known that the application of sucrose in a cryopreservation solution is beneficial for the preservation of the 3D scaffold integrity and cell viability since sucrose interacts with scaffold proteins and the cell phospholipid membranes and stabilizes them while regulating gradual cell dehydration and rehydration during freezing and thawing [77]. Indeed, our results suggest that the addition of 0.3 M sucrose to DMSO significantly improves the integrity of cell-free core-shell capsules after cryopreservation. This could be associated with the decrease of freezable water, formation of smaller ice crystals and stabilisation of hydrogel structure through hydrogen bonding. On the other hand, the presence of intracellular sucrose during cryopreservation is known to impart significant cryoprotection to suspended cells and presumably encapsulated cells. Cell loading with sucrose is commonly achieved via endocytotic uptake during prolonged pretreatment before freezing [51]. Although in this study the effect of the pretreatment of cell-free core-shell capsules 24 h before freezing was not superior for the preservation of the capsule integrity as compared to the DS group, we believe that this step is important to ensure vital cellular characteristics after cryopreservation. As can be seen from Figure 11A1–A3, we observed the disintegration and complete defragmentation of the alginate shell most frequently during freezing in 10% DMSO without sucrose. This could be attributed to the unequal thermal expansion coefficients between the frozen alginate membrane and bulk ice in an inner core as well as limited viscoelastic properties of the alginate membrane at low temperatures. Our preliminary results on analysing the viscoelastic properties could show that the cryopreservation procedures reduce the storage (G′) and loss (G′′) modulus (data not shown), suggesting that the core-shell capsules lose their elasticity after cryopreservation. Although we did not observe a positive effect of the sucrose pretreatment step on the post-thaw integrity of core-shell capsules, further optimisation studies are needed to ensure efficient preservation of their viscoelastic properties as well as functionality of 3D cellular structures.

In order to understand the possible cryodamage mechanisms, preliminary modelling of the temperature and principal stress distribution during freezing and thawing in a model core-shell capsule was conducted in COMSOL Multiphysics (COMSOL Inc., Burlington, MA, USA) utilising “Heat transfer in solids” and “Solid mechanics” modules. The detailed description of the methodology can be found in the Supplementary Materials (Figure S5). Figure 11B1,B2 shows the modelling results of the spatial temperature (B1) and principal stress (B2) distribution during cooling. As can be seen, the temperature distribution within a capsule is inhomogeneous at −20 °C, whereas the spatial thermomechanical stress increases at the shell-core interface. In contrast to the distribution profiles of the principal stress during cooling (Figure 11B3), the maximum value of principal stress during thawing does not follow a linear trend and reaches a local maximum of 60 kPa at around −120 °C (Figure 11B4), which could be interpreted by the metastable state within the glass transition region. Such an increase in the principal stress may be attributed to an unequal temperature distribution within the core-shell capsules during warming. Thus it can be hypothesised that along with the predicted thermomechanical stress increase in core-shell capsules during freezing, additional mechanical disintegration of the alginate membrane could take place during thawing and requires further analysis.

### 3.5. Future Prospects and Limitations

In summary, our results suggest the promising application of the coaxial cell encapsulation technology preparing core-shell capsules towards the practical use of encapsulated and self-organized cellular structures as well as their long-term storage. In order to improve the efficiency of the encapsulation technology and cryopreservation, the following points have to be considered:

#### 3.5.1. Application of Alginates of Clinical Grade

Although in our study we utilised a sterile-filtered solution of alginate, it could contain manufacture-related impurities which may have influenced some physical parameters of prepared core-shell capsules. To prevent any adverse effects in potential in vivo applications (fibrosis and immune response) and standardise capsule fabrication, commercially available alginates of clinical grade (but of high cost) would be a preferred option.

#### 3.5.2. Efficiency of Formation of Self-Organized Cellular Structures

In our study we observed the formation of cellular structures upon culture in around 60–70% of all core-shell capsules. This could be associated with the fact that the number of encapsulated cells per capsule decreases over the capsule production period. The cells suspended in culture medium tend to settle on the bottom of a syringe over time due to gravity resulting in a lower cell concentration in a core. This challenge could be solved technically by the application of dynamic stirring in a cell storage vessel/syringe. Therefore, future studies will analyse the effect of the cell concentration on the efficacy of cell structure formation. In the case of stirring, the arising shear stress to the cells has to be minimized.

#### 3.5.3. Preservation of the Alginate Membrane Integrity and Mechanical Properties of Formed Hydrogel Constructs after Cryopreservation

In our study we demonstrated several damaging effects which occur after thawing. This could have a number of reasons: poor penetration of the CPAs into the capsules, increased thermal stresses at the intersection of the alginate membrane with a core material, unequal temperature distribution and spontaneous ice formation at suboptimal negative temperatures. Thus, the following approaches can be considered to minimize or avoid the damaging effects occurring during freezing and thawing: modelling of the heat transfer and thermomechanical stress [78,79], application of ice-binding proteins in the alginate membrane and core material [80], induced ice formation technologies [81,82], freezing containers with improved heat transfer (such as cryobags, [83], as well as the validation of the ice-free vitrification approach [48] and radiofrequency nanowarming [84].

#### 3.5.4. Effect of Hydrogel Material Properties on the Encapsulated Cells

Stem cells are well known for their self-renewal and differentiation potential. The intricate biomechanical properties of alginate hydrogels play a significant role in controlling the phenotype, cell cytoskeleton, proliferation and differentiation capacities of the encapsulated cells. These are mainly determined by the content of the residues (M/G ratio) participating in cross-linking, type of cross-linking agent and cross-linking duration. The effect G- and M-blocks’ content on the encapsulated cells has been investigated by a number of research groups. For example, the iPSCs encapsulated within the solid beads made of high-G alginate (higher stiffness) showed prolonged exhibition of Oct4 and Nanog pluripotency markers, as compared to the beads produced from the high-M alginate (lower stiffness) [24], whereas high-M alginate promoted cell differentiation towards a primitive endoderm phenotype. In order to understand at what extent the encapsulation of cells within core-shell capsules produced form high-G and high-M alginates influence the fate of the encapsulated cells, further studies are required for a more detailed characterisation of the cells before and after encapsulation on a cellular and molecular level.

## 4. Materials and Methods

Unless otherwise stated, the chemicals were purchased from Sigma-Aldrich, Taufkirchen, Germany. Medium viscosity (Sigma-Aldrich, batch A2033, extracted from *Macrocystis pyrifera*, M/G ratio of 1.56, molecular weight 350 kDa [60], viscosity ≥ 2000 cP (2%, 25 °C)) and low viscosity (Sigma-Aldrich, batch 71238, molecular weight 100–200 kDa [61], viscosity 100–300 cP (2%, 25 °C)) alginate sodium salts from brown algae were used in the present study.

### 4.1. Preparation of Core-Shell Capsules and Solid Beads

Before the production of core-shell capsules and solid beads, the properties of the alginate solution with different concentrations, such as density, viscosity and conductivity, were tested at different temperatures. In order to evaluate the loss in viscosity during the storage at 4 °C, respective solutions of low viscosity alginate (1.5, 2.0 and 2.5%) were stored up to 34 days. Solution viscosity was evaluated using an AMVn Automated Microviscometer (Anton Paar GmbH, Graz, Austria) in the temperature range of 5–40 °C. Prior to viscosity measurements, the temperature-dependent solution density was measured using a DMA 38 Density Meter (Anton Paar GmbH, Graz, Austria). The electrical conductivity of the alginate solutions was measured using a WTW Multi250i device with WTW OxiCal CX measuring probe (Xylem Analytics, Weilheim, Germany) in the temperature range of 3–38 °C.

**Figure 12:**
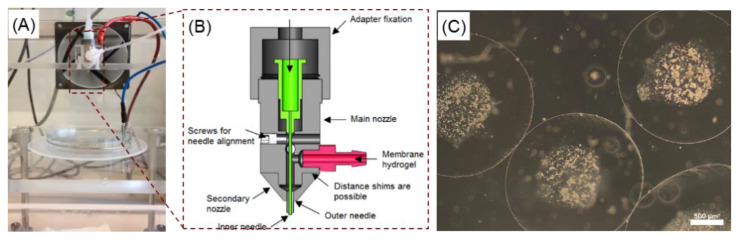
Coaxial encapsulation of cells using electrospraying. (**A**) General view of the set-up used for coaxial encapsulation. (**B**) Custom-developed coaxial nozzle. (**C**) Core-shell capsules laden with *cj*aMSCs (cell encapsulation density 5 × 10^6^ cells/mL). Scale bar is 500 µm.

The production of cell-free and cell-laden core-shell capsules as well as solid beads was conducted using the electrospraying approach modified by Gryshkov et al. [30]. The general process of electrospraying included the pumping of the alginate solution and/or cell suspension at defined flow rates through a coaxial nozzle, application of a high voltage between a nozzle and gelling solution and transportation of the produced alginate droplets to a bath containing a gelling solution of 100 mM calcium chloride (CaCl_2_, Carl Roth, Karlsruhe, Germany) for cross-linking. For the core-shell capsules and solid beads, sodium alginates were dissolved in 10 mM HEPES (pH 7.4, Carl Roth, Karlsruhe, Germany) and 0.9% sodium chloride. Before the bead preparation, the alginate solutions were sterile filtered through a 0.22 µm filter (TPP, Trasadingen, Switzerland). In the case of core-shell capsules, the membrane (alginate) and core (HEPES or cell suspension in culture medium) solutions were pumped simultaneously at different flow rates through the custom-developed coaxial nozzle shown in Figure 12A,B. After the respective gelling time alginate beads were collected and washed once with a washing solution (20 mM CaCl_2_ in 10 mM HEPES containing 0.9% NaCl (pH 7.4)) followed by a final wash in 10 mM HEPES and used for further analysis.

Table 2 summarises the main parameters used for the production of core-shell capsules and the solid beads for the tests and analyses used in this study.

In order to analyse the effect of the electrospraying approach on the size of the core-shell capsules, the applied high voltage (0–17.5 kV) and ratio of the flow rates of the alginate to the inner fluid (HEPES, 4:2 to 14:2) were varied. The following parameters for the production of solid beads were used: needle 21G (outer diameter 0.8 mm), 100 mM CaCl_2_ gelling solution, gelling time 30 min, spraying distance 10 cm, applied voltage 7.5 kV, alginate flow rate 10 mL/h. Using our electrospraying setup configuration, a combination of these parameters yields in the production of solid beads possessing a diameter of 3000 ± 200 µm.

For the production of the core-shell capsules, the alginate and HEPES (or cell suspension) solutions were pumped through a 14G outer (outer diameter 1.83 mm) and 27G (outer diameter 0.4 mm) inner needle using two syringe pumps (KD Scientific Inc., Holliston, MA, USA). A coaxial nozzle was charged positively, whereas an electrode was grounded and immersed into the gelling solution. The distance between the tip of the outer needle and the surface of the gelling solution represents the spraying distance. Measurements of the outer diameter of the core-shell capsules, membrane thickness and diameter of the inner core were performed using an AxioVert. A1 microscope and ZEN Blue software (Carl Zeiss, Jena, Germany).

### 4.2. RAMAN Spectroscopy, Water Content and Swelling Behaviour

The core-shell capsules and solid beads used for the analysis of the structural composition (RAMAN spectroscopy), water content and swelling behaviour possessed the following dimensions: (i) LV alginate core-shell capsules—outer diameter 3046 ± 56 µm, inner diameter 1675 ± 43 µm; (ii) LV alginate solid beads—diameter 3044 ± 47 µm; (iii) MV alginate core-shell capsules—outer diameter 3215 ± 59 µm, inner diameter 1941 ± 88 µm; (iv) MV alginate solid beads—diameter 3115 ± 48 µm.

RAMAN spectroscopy was used to analyse the structure (cross-linking and M/G content) of the core-shell capsules after the production and freeze-drying (dried materials). The freeze-drying step was conducted in an Epsilon 2–10 D (Martin Christ, Osterode am Harz, Germany) freeze-drying system (freezing to −30 °C, primary drying at 0.340 mPa and −30 °C for 10 h followed by a final drying at 0.009 mPa and 20 °C for 10 h). The RAMAN spectra of hydrogels and dried materials were compared with each other and the spectra of the respective powders used for the preparation of the materials. Spectral acquisition was conducted using an alpha300RA microscope (WiTec GmbH, Ulm, Germany) in the wavenumber range of 200–1800 cm^−1^ (fingerprint region). The microscope was controlled using the Control FIVE.plus software (WiTec GmbH, Ulm, Germany). Prior to measurement, a Si wafer was used to calibrate the system with the Si peak at 520 cm^−1^, which corresponds to the crystalline Si-Si bond longitudinal optical phonon vibrations). The 532 nm laser (10 mW, TruePower-controlled) was focused on a sample using a 10x Carl Zeiss objective to excite molecular vibrations. An Ultra High Throughput Spectrometer (UHTS300) with a grating of 600 lines/mm and a Peltier-cooled CCD camera (−60 °C working temperature, 600 groves/mm) was used for emission detection. The spectra were collected using 5 s integration time and a total number of 20 accumulations. After collection of the spectra, they were processed within the Project FIVE.plus software (WiTec GmbH, Ulm, Germany) by applying cosmic-ray removal (filter size 2, dynamic factor 2), smoothing with a Savitzky-Golay (points 5, polynomial order 2) followed by the background subtraction using a shape function (shape size 250, noise factor 3. The spectra were normalised using the Equation (1):

(1) $$I_{norm}=\frac{I_{y}-I_{\min}}{I_{\max}-I_{\min}},$$

where *I_norm_* is the normalised intensity, *I_max_* is the maximum intensity value, *I_min_* is the minimum intensity value and *I_y_* is the measured intensity [arb. un.].

In order to analyse the storage stability of the produced core-shell capsules and solid beads, they were incubated in WS at 4 °C for 7 day. Afterwards, the core-shell capsules and the solid beads were distributed within the wells of 6-well culture plates with 6 capsules/beads per well followed by the addition of four different solutions: 0.9% (*w*/*v*) sodium chloride (NaCl), HEPES, FBS-free basal medium and FBS-containing complete cell culture medium (all having pH 7.4). The capsules/beads were then incubated in a 37 °C incubator with 5% CO_2_ with a solution exchange on the day of the analysis. The respective dimensions of the core-shell capsules and the solid beads were measured before swelling (day 0) and on days 1, 4 and 7 upon swelling. In total, the sizes of 6 capsules/beads were measured in triplicates (*n* = 18). In addition, the osmolality of the solutions measured using an Osmomat 030 device (Gonotec GmbH, Berlin, Germany).

For the evaluation of the water uptake and water content, the weight of the produced core-shell capsules and solid beads was measured before (wet) and after freeze-drying (freeze-dried), as detailed above. In total, the weight of 90 capsules/beads per condition was measured (*n* = 90). The water uptake was calculated using the Equation (2)

(2) $$W_{u}[\%]=\frac{m_{wet}-m_{dry}}{m_{wet}}\times 100\%,$$

where *W_u_* is the water uptake in %, *m_we_*_t_ and *m_dry_* are the respective weights of the capsules before and after freeze-drying in mg.

In turn, the water content was calculated according to the following Equation (3):

(3) $$W_{c}=\frac{m_{wet}-m_{dry}}{m_{dry}},$$

where *W_c_* is the content of water per mg of the freeze-dried material in mg_water_/mg_dry_.

### 4.3. Cell Culture and Encapsulation

In this work, two types of MSCs were used for the cell encapsulation: amnion MSCs derived from the placenta of the common marmoset *Callithrix Jacchus* (*cj*aMSCs, all cell studies except for metabolic activity) and human dermal MSCs (hdMSCs, metabolic activity). The latter investigations were performed by the cooperation partners from the Institute for Problems of Cryobiology and Cryomedicine of the National Academy of Sciences of Ukraine for an independent validation of the coaxial encapsulation in a scientific facility different from the corresponding author.

The phenotypic characterisation of hdMSCs and *cj*aMSCs used throughout the experiments was performed by fluorescence-activated cell sorting. It was shown that more than 90% of hdMSCs expressed CD29, CD73, CD90, and CD105, but were negative for CD34 and CD45 [51]. After induction of differentiation, isolated cells underwent adipogenic, osteogenic and chondrogenic transformation in vitro. The ability to multilineage differentiation of hdMSCs released from solid beads has previously been shown [72]. The *cj*aMSCs have also been characterised on cellular and molecular level [33,85].

The *cj*aMSCs were cultivated under sterile conditions in Dulbecco’s modified Eagle’s medium (DMEM, batch 9007.1, Carl Roth, Karlsruhe, Germany) containing 15% (*v*/*v*) fetal bovine serum (Biochrom GmbH, Berlin, Germany), 1% penicillin/streptomycin, 1% ascorbic acid in 10 cm tissue culture dishes (TPP, Trasadingen, Switzerland) in a humidified incubator at 37 °C and 5% CO_2_ until 70% confluency. For encapsulation, the cells were harvested using 0.05%/0.02% (*w*/*v*) trypsin/EDTA solution for 4 min and pelleted using centrifugation at 1000 rpm for 5 min. Before encapsulation, the cell membrane integrity was analysed using a Vi-CELL XR Cell Viability Analyzer (Beckman Coulter GmbH, Krefeld, Germany). In all cell encapsulation experiments, the cell membrane integrity was above 95%. After counting, the cells were resuspended in cell culture medium (for core-shell capsules) or in 10 mM sterile HEPES solution (solid beads) yielding in a final concentration of 5 × 10^6^ cells/mL.

Cultures of hdMSCs were maintained in T25 adhesive polystyrene cell culture flasks (TPP, Trasadingen, Switzerland) at 37 °C, 5% CO_2_, and 95% humidity in α-MEM (Minimum Essential Medium Eagle—alpha modification) containing 10% FBS (Biowest, Nuaillé, France), 50 μg/mL penicillin (Biowest, Nuaillé, France), 50 μg/mL streptomycin (Biowest, Nuaillé, France), and 0.2 mM l-glutamine. Before encapsulation, they were pelleted by the centrifugation at 150× *g* for 5 min. Before cell encapsulation, all parts of the electrospraying device (apart from single-use sterile consumables) were disinfected with Bacillol AF (BODE Chemie GmbH, Hamburg, Germany), whereas a coaxial nozzle (for core-shell capsules) and grounded electrode (stainless steel ring) were autoclaved. The utilised process parameters for the preparation of cell-laden core-shell capsules and solid beads can be consulted in the Table 2 of the Section 4.1. After 30 min of gelling both, the core-shell and solid beads were washed with sterile washing and HEPES solutions and used for further experiments. The MSCs of passages 6–9 were used in the studies. Knowing the initial cell concentration for encapsulation (5 × 10^6^ cells/mL) and a mean volume of a core (3 × 10^−3^ mL), the average cell number per a single core-shell capsule could be calculated as being 15 × 10^3^ cells.

The human embryonic stem cells (HES-3 cell line) grew in typical flat-type round colonies on Matrigel™-coated cell culture plastic, with individual cells presenting a high nucleus to cytoplasm ratio. Flow cytometry analysis showed expression of typical pluripotency-associated antigens such as stage-specific embryonic antigen 4 (SSEA4) and tumor rejection antigens TRA-1-60 and TRA-1-81. Moreover, hESC cells of the HES-3 line depicted a strong expression of the pluripotency master regulators POU5F1 (Oct4), SOX2 on the mRNA and protein level. Upon withdrawal of pluripotency-maintaining supplements in the culture medium, spontaneous differentiation was observed. Directed differentiation into the two germ layers definitive endoderm and mesoderm was shown [86].

### 4.4. Cryopreservation

In order to pre-select cryopreservation parameters to protect both the structure of the core-shell capsules and hence encapsulated cells, a parametric study involving cell-free capsules has been conducted. For this, we evaluated the effect of the incubation time of the cell-free core-shell capsules with CPAs for 15 and 45 min, using the conventional and modified “in air” cryopreservation approach as well as a type of CPA solution. Among the CPAs, we analysed the effect of commonly used 10% DMSO, 10% DMSO with 0.3 M sucrose as well as the pretreatment of core-shell capsules with 0.1 M sucrose for 24 h followed by the freezing in the cryoprotective solution composed of 10% DMSO and 0.3 M sucrose.

#### 4.4.1. Cryopreservation of Cell-Free Core-Shell Capsules

For the validation of the cell-free cryopreservation, the core-shell capsules having an outer diameter of 3000 ± 50 µm, an inner diameter of 1600 ± 60 µm and a membrane thickness of around 700 µm were used. After gelling for 30 min, one set of capsules was pretreated with 0.1 M sucrose. Another set of cell-free core-shell capsules (control) was cultivated in an incubator for 24 h within the cell culture medium. After incubation, pretreated and not pretreated cell-free core-shell capsules were transferred into cryovials (TPP, Trasadingen, Switzerland) with 20 capsules per vial and placed on ice for CPA loading. The following cryopreservation groups were compared: 0% DMSO (negative control, without CPAs), 10% DMSO (D), 10% DMSO with 0.3 M sucrose (DS) and 10% DMSO with 0.3 M sucrose pretreated with 0.1 M sucrose (DSPT). The freezing solutions were prepared in a pure cell culture medium supplemented with FBS in a final concentration of 20% (*v*/*v*). Loading with CPAs was conducted on ice for 15 and 45 min. After loading, capsules were cryopreserved using two protocols. The common steps were freezing at 1 K/min to −100 °C in the controlled rate freezer Planer Kryo 560-16 (Planer Limited, Middlesex, United Kingdom) and storage (at least 3 days) in liquid nitrogen. In the procedure referred herein as to ‘conventional’ samples were frozen in a bulk (1 mL) CPA solution and thawed in a 37 °C water bath with gentle shaking for around 2 min. In contrast, in the ‘modified’ procedure the CPA solution was aspirated after equilibration of the samples and two-step thawing was performed: thawing for 30 s in a 37 °C water bath followed by the fast addition of 37 °C pre-warmed cell culture medium.

**Figure 13:**
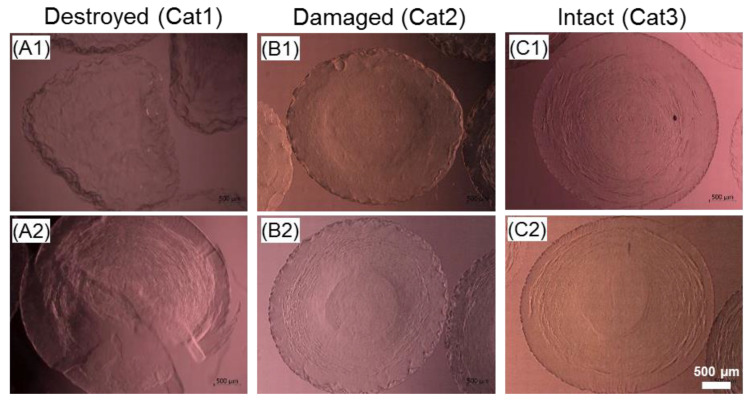
Gradation of damaging effects of core-shell capsules after thawing. (**A**) Destroyed capsules include (**A1**) a shrunken sample with the completely lost shape and (**A2**) a sample with completely disintegrated membrane. (**B**) Core-shell capsules with moderately (**B1**) and severely damaged membranes (**B2**). (**C**) Intact core-shell capsules possessing an intact outer layer, minor membrane damage, hardly visible (**C1**) and good visible (**C2**) core. Scale bars are 500 µm.

After thawing, capsules were transferred into 6-well culture plates (TPP, Trasadingen, Switzerland) and used for further analysis. Samples were subjected to microscopic examination in order to calculate the outer and inner diameters as well as the membrane thickness. The data referring to the capsule integrity were derived according to the gradation of damaging effects to the alginate membrane as shown in Figure 13: destroyed (A, Category 3), damaged (B, Category 3) and intact (C, Category 3). The core-shell capsules with the characteristics defined in the Category 3 were considered as intact and used for further validation of the cryopreservation for cell-laden samples.

#### 4.4.2. Cryopreservation of Cell-Laden Core-Shell Capsules

In all cryopreservation studies involving the *cj*aMSCs, the core-shell capsules having an outer diameter of around 3500 µm and a core diameter of around 1800 µm were used. The cryopreservation of core-shell capsules containing self-organized cellular constructs was conducted on day 35 of culture using the CPA type and loading time pre-selected from the parametric study involving cell-free core-shell capsules. This included the application of 10% DMSO, 0.3 M sucrose, an additional pretreatment step with 0.1 M sucrose before freezing and CPA loading for 45 min. The cell-laden core-shell capsules were cryopreserved using conventional and modified approaches as described in the Section 4.4.1. Storage and thawing processes were performed in accordance with the pre-selected parameters resulted from the cryopreservation of cell-free capsules, unless stated otherwise. After thawing (20 core-shell capsules per cryovial), cell-laden core-shell capsules were re-cultivated for 7 days with a regular medium exchange. After re-cultivation, cell viability was analysed using live-dead staining described below.

### 4.5. Analysis of the Cell Viability, Metabolic Activity, Cell–Cell and Cell-Scaffold Interactions

The viability of the cells encapsulated in solid beads and core-shell capsules was assessed using a live-dead assay before encapsulation, before cryopreservation (after culture for 35 days) and after thawing. For that, staining of samples with fluorescent dyes Calcein AM (excitation/emission: 494/517 nm) and Ethidium Homodimer-1 (excitation/emission: 528/617 nm) (CaAM/EthD-1) was performed. The staining solution of 2 µM Calcein AM was prepared directly from 1 mM stock solution in anhydrous DMSO (Biotium, Fremont, CA, USA) in 10 mM HEPES, whereas the 4 µM EthD-1 staining solution was prepared from a 1 mM stock solution in 10 mM HEPES. The 1 mM EthD-1 stock solution was in turn prepared by dissolving 1 mg of Ethidium Homodimer powder in 1.167 mL DMSO.

The general appearance of the cell cytoskeleton and cell–cell interactions within the 3D cellular networks were analysed using a Phalloidin (stains actin filaments in green) and Hoechst (stain cell nuclei in blue) fluorescence-based staining. Before staining, the capsules were fixed with 4% formaldehyde for 30 min and the cell membranes were permeabilised using 0.1% TritonX-100 solution by incubating for 15 min followed by a single washing step in phosphate-buffered saline (PBS, Biochrom GmbH, Berlin, Germany). Next, cell-laden core-shell capsules were incubated in 2 µM Phalloidin (excitation/emission: 495/520 nm) and 1 µM Hoechst33342 (excitation/emission: 346/460 nm) staining solution prepared in PBS for 30 min in the dark. After washing, image acquisition for both fluorescent assays was conducted using a motorized Axiovert 200M fluorescence microscope (Carl Zeiss, Jena, Germany) and AxioVision software (Rel. 4.8).

The metabolic activity of hdMSCs within the core-shell capsules and solid beads was assessed using an Alamar Blue assay (AB, Serotec Ltd., Oxford, UK). Cells were encapsulated at a concentration of 1.5 × 10^6^ cells/mL. After encapsulation, samples were cultured in a humidified incubator at 37 °C and 5% CO_2_. On day 1, 3 and 5 AB solution was added to the culture medium in a final concentration of 10% (*v*/*v*). After incubation for 2 h the reduced AB solution was collected by medium exchange and fluorescence was analysed at 550 nm (excitation) and 590 nm (emission) by a TECAN GENios microplate reader (Tecan Genios; Tecan, Austria). The obtained data were analysed using the XFluor4 software and presented as Relative Fluorescent Units (RFUs) per well. RFUs were calculated as (*A_sample_*—*A_blank_*)/*A_blank_*, where *A_sample_* are the fluorescence values of reduced AB and *A_blank_*—fluorescence values of 10% AB without cells.

For histological examinations, the core-shell capsules containing self-organized cellular structures formed by *cj*aMSCs on day 35 of culture were fixed in 3.7% formaldehyde for 24 h and dehydrated in increasing concentrations of ethanol (50%, 70%, 96% and 100% (*v*/*v*)) every time for 4 h. Afterwards, the samples were cleared in 100% xylene for 4 h and embedded in Technovit 7100 (Heraeus Kulzer, Hanau, Germany). Sections with a thickness of 5 µm were produced using a rotary microtome HM 355 (Microm, Walldorf, Germany). All the sections were mounted using DPX medium (Merck, Darmstadt, Germany) and stained with 0.1% toluidine blue solution for the subsequent imaging.

### 4.6. Statistical Analysis

All statistical analyses were performed in OriginPro 2021 (OriginLab Corporation, Northampton, MA, USA). The normality of data was assessed by applying a Shapiro–Wilk test. If normal, statistical significances were analysed using one-way ANOVA (*p* = 0.05) followed by a Tukey’s multiple comparison test and the data were presented as a mean and standard deviation. Not normally distributed values were analysed using the non-parametric Kruskal–Wallis-test and post hoc Dunn’s multiple comparison test (*p* = 0.05). The values at *p* < 0.05 were considered as significantly different.

## Figures and Tables

**Table 1:** The results on analysing the water uptake and content of fifteen core-shell capsules and solid beads.

Property	Core-Shell Capsules (*n* = 15)		Solid Beads (*n* = 15)	
	LV	MV	LV	MV
Hydrogel weight *, mg	175.8 ± 2.7	225.7 ± 2.5	180.0 ± 12.2	212.6 ± 2.5
Dry weight *, mg	6.4 ± 0.3	9.3 ± 0.3	7.6 ± 0.3	10.4 ± 0.2
Water uptake (*W_u_*), %	96.4 ± 0.2	95.9 ± 0.2	95.8 ± 0.4	95.1 ± 0.1
Water content (*W_c_*), mg_water_/mg_dry_	26.7 ± 1.4	23.4 ± 0.9	22.7 ± 2.1	19.5 ± 0.3

* error of the scales is 0.1 mg.

**Table 2:** Summary of process parameters used for solution characterisation and preparation of core-shell capsules and solid beads.

Test/Analysis	Alginate Concentration and Type, %	Type of Nozzles (Inner—Outer)	Voltage, kV	Spraying Distance, cm	Inner to Outer Nozzle Distance, mm	Flow Rates (Outer:Inner), mL/h:mL/h	Gelling Time, min
Density, viscosity, conductivity	1.5% LV 2.0% LV	-	-	-	-	-	-
Main optimisation studies *	1.5% LV caps	21G–14G	0–17.5	5–10	0–1.0	4:2–50:10	30
	2.0% LV caps	21G–14G			0–1.0	4:2–50:10	
	2.0% LV beads	14G			-		
	2.0% MV caps	21G–14G			0–1.0	4:2–50:10	
	2.0% MV beads	14G			-		
RAMAN spectroscopy, water content, swelling	2.0% LV caps	21G–14G	9.5	5	0.5	20:4	30
	2.0% LV beads	14G	9.5	5	-	20	
	2.0% MV caps	21G–14G	6.5	5	0.5	20:4	
	2.0% MV beads	14G	6.5	5	-	20	
Fluorescent staining (*cj*aMSC-laden)	2.0% LV caps	21G–14G	12	10	0.5	50:15	30
	2.0% LV beads	14G	12	10	-	50	
	2.0% MV caps	21G–14G	10	10	0.5	50:10	
Cryopreservation (cell-free)	2.0% LV caps 2.0% MV caps	21G–14G	10	5	0.5	40:8	30
Histology, cryopreservation (cell-laden)	2.0% LV caps	21G–14G	0	5	0.5	40:8	30
Metabolic activity (hdMSC-laden)	2.0% LV caps	21G–14G	7	4	0.5	40:10	15
	2.0% LV beads	14G	7	4	-	40	15
hESCs encapsulation	1.5% LV caps	21G–14G	18	10	0.5	40:10	30

LV—low viscosity; MV—medium viscosity; %—*w*/*v*; caps—core-shell capsules; beads—solid beads; *cj*aMSCs—*Callithrix Jacchus* amnion-derived multipotent stromal cells; hdMSCs—human dermal multipotent stromal cells; hESCs—human embryonic stem cell. * for the main optimisation studies the following constant process parameters were applied: distance between the inner and outer nozzle 0.5 mm, the spraying distance 7.5 cm, the ratio of alginate to inner fluid flow rates 14:2 and the applied voltage 10 kV.

## Data Availability

All reported data that support the findings of this study are in the manuscript and Supplementary Materials. The raw data are available from the corresponding author upon reasonable request.

## Supplementary Materials

The following are available online at https://www.mdpi.com/1422-0067/22/6/3096/s1, Figure S1: Effect of the alginate concentration and temperature on the density (A), viscosity (B) and electrical conductivity (C) of the LV alginate solution; Figure S2: Bright-field images of core-shell capsules after cryopreservation; Figure S3: Effect of the absolute value of flow rates of the inner fluid and alginate ((A), efficiency of scaling up) and alginate concentration (B) on the size of the alginate core-shell capsules; Figure S4: Representative fluorescent images of *cj*aMSCs (35 days in culture) within core-shell capsules produced from MV alginate; Figure S5: Geometry of the heat transfer model. References [87,88,89,90,91] are cited in the supplementary materials.

## References

[1] Leslie S.K., Kinney R.C., Schwartz Z., Boyan B.D. (2017). Microencapsulation of Stem Cells for Therapy. Methods Mol. Biol..

[2] Ahmad Raus R., Wan Nawawi W.M.F., Nasaruddin R.R. (2020). Alginate and alginate composites for biomedical applications. Asian J. Pharm. Sci..

[3] Kaczmarek-Pawelska A., Pereira L. (2020). Alginate-Based Hydrogels in Regenerative Medicine. Alginates—Recent Uses of This Natural Polymer.

[4] Aderibigbe B.A., Buyana B. (2018). Alginate in Wound Dressings. Pharmaceutics.

[5] Cao L., Lu W., Mata A., Nishinari K., Fang Y. (2020). Egg-box model-based gelation of alginate and pectin: A review. Carbohydr. Polym..

[6] Pawar S.N., Edgar K.J. (2012). Alginate derivatization: A review of chemistry, properties and applications. Biomaterials.

[7] Mørch Y.A., Donati I., Strand B.L., Skjåk-Braek G. (2006). Effect of Ca^2+^, Ba^2+^, and Sr^2+^ on alginate microbeads. Biomacromolecules.

[8] Donati I., Holtan S., Mørch Y.A., Borgogna M., Dentini M., Skjåk-Braek G. (2005). New hypothesis on the role of alternating sequences in calcium-alginate gels. Biomacromolecules.

[9] Abasalizadeh F., Moghaddam S.V., Alizadeh E., Akbari E., Kashani E., Fazljou S.M.B., Torbati M., Akbarzadeh A. (2020). Alginate-based hydrogels as drug delivery vehicles in cancer treatment and their applications in wound dressing and 3D bioprinting. J. Biol. Eng..

[10] Sarker B., Boccaccini A.R., Rehm B.H.A., Moradali M.F. (2018). Alginate Utilization in Tissue Engineering and Cell Therapy. Alginates and Their Biomedical Applications.

[11] Pravdyuk A.I., Petrenko Y.A., Fuller B.J., Petrenko A.Y. (2013). Cryopreservation of alginate encapsulated mesenchymal stromal cells. Cryobiology.

[12] Sahoo D.R., Biswal T. (2021). Alginate and its application to tissue engineering. SN Appl. Sci..

[13] Serrano-Aroca Á., Vera-Donoso C.D., Moreno-Manzano V. (2018). Bioengineering Approaches for Bladder Regeneration. Int. J. Mol. Sci..

[14] Venkatesan J., Bhatnagar I., Manivasagan P., Kang K.-H., Kim S.-K. (2015). Alginate composites for bone tissue engineering: A review. Int. J. Biol. Macromol..

[15] Zhang C., Zhou Y., Zhang L., Wu L., Chen Y., Xie D., Chen W. (2018). Hydrogel Cryopreservation System: An Effective Method for Cell Storage. Int. J. Mol. Sci..

[16] Smidsrød O., Skjåk-Braek G. (1990). Alginate as immobilization matrix for cells. Trends Biotechnol..

[17] Bochenek M.A., Veiseh O., Vegas A.J., McGarrigle J.J., Qi M., Marchese E., Omami M., Doloff J.C., Mendoza-Elias J., Nourmohammadzadeh M. (2018). Alginate encapsulation as long-term immune protection of allogeneic pancreatic islet cells transplanted into the omental bursa of macaques. Nat. Biomed. Eng..

[18] Bhujbal S.V., Paredes-Juarez G.A., Niclou S.P., De Vos P. (2014). Factors influencing the mechanical stability of alginate beads applicable for immunoisolation of mammalian cells. J. Mech. Behav. Biomed. Mater..

[19] de Vos P., Faas M.M., Strand B., Calafiore R. (2006). Alginate-based microcapsules for immunoisolation of pancreatic islets. Biomaterials.

[20] Safley S.A., Cui H., Cauffiel S., Tucker-Burden C., Weber C.J. (2008). Biocompatibility and immune acceptance of adult porcine islets transplanted intraperitoneally in diabetic NOD mice in calcium alginate poly-L-lysine microcapsules versus barium alginate microcapsules without poly-L-lysine. J. Diabetes Sci. Technol..

[21] Köllmer M., Appel A.A., Somo S.I., Brey E.M. (2016). Long-Term Function of Alginate-Encapsulated Islets. Tissue Eng. Part B Rev..

[22] Selden C., Bundy J., Erro E., Puschmann E., Miller M., Kahn D., Hodgson H., Fuller B., Gonzalez-Molina J., Le Lay A. (2017). A clinical-scale BioArtificial Liver, developed for GMP, improved clinical parameters of liver function in porcine liver failure. Sci. Rep..

[23] Qi M., Strand B.L., Mørch Y., Lacík I., Wang Y., Salehi P., Barbaro B., Gangemi A., Kuechle J., Romagnoli T. (2008). Encapsulation of human islets in novel inhomogeneous alginate-ca2+/ba2+ microbeads: In vitro and in vivo function. Artif. Cells Blood Substit. Immobil. Biotechnol..

[24] Wilson J.L., Najia M.A., Saeed R., McDevitt T.C. (2014). Alginate encapsulation parameters influence the differentiation of microencapsulated embryonic stem cell aggregates. Biotechnol. Bioeng..

[25] Tang H.-C., Chen W.-C., Chiang C.-W., Chen L.-Y., Chang Y.-C., Chen C.-H. (2015). Differentiation Effects of Platelet-Rich Plasma Concentrations on Synovial Fluid Mesenchymal Stem Cells from Pigs Cultivated in Alginate Complex Hydrogel. Int. J. Mol. Sci..

[26] Chayosumrit M., Tuch B., Sidhu K. (2010). Alginate microcapsule for propagation and directed differentiation of hESCs to definitive endoderm. Biomaterials.

[27] Kim W.S., Mooney D.J., Arany P.R., Lee K., Huebsch N., Kim J. (2012). Adipose tissue engineering using injectable, oxidized alginate hydrogels. Tissue Eng. Part A.

[28] Bidarra S.J., Torres A.L., Barrias C.C. (2016). Injectable Cell Delivery Systems Based on Alginate Hydrogels for Regenerative Therapies. Reference Module in Materials Science and Materials Engineering.

[29] Kang A., Park J., Ju J., Jeong G.S., Lee S.-H. (2014). Cell encapsulation via microtechnologies. Biomaterials.

[30] Gryshkov O., Pogozhykh D., Zernetsch H., Hofmann N., Mueller T., Glasmacher B. (2014). Process engineering of high voltage alginate encapsulation of mesenchymal stem cells. Mater. Sci. Eng. C Mater. Biol. Appl..

[31] Sharma V., Hunckler M., Ramasubramanian M.K., Opara E.C., Katuri K.C. (2017). Microfluidic Approach to Cell Microencapsulation. Methods Mol. Biol..

[32] Prüsse U., Bilancetti L., Bučko M., Bugarski B., Bukowski J., Gemeiner P., Lewińska D., Manojlovic V., Massart B., Nastruzzi C. (2008). Comparison of different technologies for alginate beads production. Chem. Pap..

[33] Gryshkov O., Pogozhykh D., Hofmann N., Pogozhykh O., Mueller T., Glasmacher B. (2014). Encapsulating non-human primate multipotent stromal cells in alginate via high voltage for cell-based therapies and cryopreservation. PLoS ONE.

[34] Hu M., Zheng G., Zhao D., Yu W. (2020). Characterization of the structure and diffusion behavior of calcium alginate gel beads. J. Appl. Polym. Sci..

[35] Veiseh O., Doloff J.C., Ma M., Vegas A.J., Tam H.H., Bader A.R., Li J., Langan E., Wyckoff J., Loo W.S. (2015). Size- and shape-dependent foreign body immune response to materials implanted in rodents and non-human primates. Nat. Mater..

[36] Workman V.L., Dunnett S.B., Kille P., Palmer D.D. (2007). Microfluidic chip-based synthesis of alginate microspheres for encapsulation of immortalized human cells. Biomicrofluidics.

[37] Horiguchi I., Sakai Y. (2015). Alginate Encapsulation of Pluripotent Stem Cells Using a Co-axial Nozzle. J. Vis. Exp..

[38] Urbani L., Maghsoudlou P., Milan A., Menikou M., Hagen C.K., Totonelli G., Camilli C., Eaton S., Burns A., Olivo A. (2017). Long-term cryopreservation of decellularised oesophagi for tissue engineering clinical application. PLoS ONE.

[39] Wang Z., Qin T. (2013). Review: Vitreous Cryopreservation of Tissue-engineered Compositions for Tissue Repair. J. Med. Biol. Eng..

[40] Shi Q., Xie Y., Wang Y., Li S. (2017). Vitrification versus slow freezing for human ovarian tissue cryopreservation: A systematic review and meta-anlaysis. Sci. Rep..

[41] Leonel E.C.R., Corral A., Risco R., Camboni A., Taboga S.R., Kilbride P., Vazquez M., Morris J., Dolmans M.-M., Amorim C.A. (2019). Stepped vitrification technique for human ovarian tissue cryopreservation. Sci. Rep..

[42] Pegg D.E. (2010). The relevance of ice crystal formation for the cryopreservation of tissues and organs. Cryobiology.

[43] Huang H., Choi J.K., Rao W., Zhao S., Agarwal P., Zhao G., He X. (2015). Alginate Hydrogel Microencapsulation Inhibits Devitrification and Enables Large-Volume Low-CPA Cell Vitrification. Adv. Funct. Mater..

[44] Camboni A., van Langendonckt A., Donnez J., Vanacker J., Dolmans M.M., Amorim C.A. (2013). Alginate beads as a tool to handle, cryopreserve and culture isolated human primordial/primary follicles. Cryobiology.

[45] Pogozhykh O., Prokopyuk V., Prokopyuk O., Kuleshova L., Goltsev A., Figueiredo C., Pogozhykh D. (2018). Towards biobanking technologies for natural and bioengineered multicellular placental constructs. Biomaterials.

[46] Serra M., Correia C., Malpique R., Brito C., Jensen J., Bjorquist P., Carrondo M.J.T., Alves P.M. (2011). Microencapsulation technology: A powerful tool for integrating expansion and cryopreservation of human embryonic stem cells. PLoS ONE.

[47] Lu Y.-C., Fu D.-J., An D., Chiu A., Schwartz R., Nikitin A.Y., Ma M. (2017). Scalable Production and Cryostorage of Organoids Using Core-Shell Decoupled Hydrogel Capsules. Adv. Biosyst..

[48] Zhao G., Liu X., Zhu K., He X. (2017). Hydrogel Encapsulation Facilitates Rapid-Cooling Cryopreservation of Stem Cell-Laden Core-Shell Microcapsules as Cell-Biomaterial Constructs. Adv. Healthc. Mater..

[49] Heinemann M., Meinberg H., Büchs J., Koss H.-J., Ansorge-Schumacher M.B. (2005). Method for quantitative determination of spatial polymer distribution in alginate beads using Raman spectroscopy. Appl. Spectrosc..

[50] Schmid T., Messmer A., Yeo B.-S., Zhang W., Zenobi R. (2008). Towards chemical analysis of nanostructures in biofilms II: Tip-enhanced Raman spectroscopy of alginates. Anal. Bioanal. Chem..

[51] Petrenko Y.A., Rogulska O.Y., Mutsenko V.V., Petrenko A.Y. (2014). A sugar pretreatment as a new approach to the Me2SO- and xeno-free cryopreservation of human mesenchymal stromal cells. CryoLetters.

[52] Mutsenko V., Knaack S., Lauterboeck L., Tarusin D., Sydykov B., Cabiscol R., Ivnev D., Belikan J., Beck A., Dipresa D. (2020). Effect of ‘in air’ freezing on post-thaw recovery of Callithrix jacchus mesenchymal stromal cells and properties of 3D collagen-hydroxyapatite scaffolds. Cryobiology.

[53] Claverie M., McReynolds C., Petitpas A., Thomas M., Fernandes S.C.M. (2020). Marine-Derived Polymeric Materials and Biomimetics: An Overview. Polymers.

[54] Gheorghita Puscaselu R., Lobiuc A., Dimian M., Covasa M. (2020). Alginate: From Food Industry to Biomedical Applications and Management of Metabolic Disorders. Polymers.

[55] Gu F., Amsden B., Neufeld R. (2004). Sustained delivery of vascular endothelial growth factor with alginate beads. J. Control. Release.

[56] Hariyadi D.M., Islam N. (2020). Current Status of Alginate in Drug Delivery. Adv. Pharmacol. Pharm. Sci..

[57] Zhang W., He X. (2009). Encapsulation of living cells in small (approximately 100 microm) alginate microcapsules by electrostatic spraying: A parametric study. J. Biomech. Eng..

[58] Salomonsen T., Jensen H.M., Stenbæk D., Engelsen S.B. (2008). Chemometric prediction of alginate monomer composition: A comparative spectroscopic study using IR, Raman, NIR and NMR. Carbohydr. Polym..

[59] Pielesz A., Bak M.K.K. (2008). Raman spectroscopy and WAXS method as a tool for analysing ion-exchange properties of alginate hydrogels. Int. J. Biol. Macromol..

[60] Pamies R., Schmidt R.R., Martínez M.d.C.L., La Torre J.G.d. (2010). The influence of mono and divalent cations on dilute and non-dilute aqueous solutions of sodium alginates. Carbohydr. Polym..

[61] Bruchet M., Mendelson N., Melman A. (2013). Photochemical Patterning of Ionically Cross-Linked Hydrogels. Processes.

[62] Stokke B.T., Smidsrød O., Zanetti F., Strand W., Skjåk-Bræk G. (1993). Distribution of uronate residues in alginate chains in relation to alginate gelling properties—2: Enrichment of β-d-mannuronic acid and depletion of α-l-guluronic acid in sol fraction. Carbohydr. Polym..

[63] Draget K.I., Strand B., Hartmann M., Valla S., Smidsrød O., Skjåk-Bræk G. (2000). Ionic and acid gel formation of epimerised alginates; the effect of AlgE4. Int. J. Biol. Macromol..

[64] Chandía N. (2001). Alginic acids in Lessonia trabeculata: Characterization by formic acid hydrolysis and FT-IR spectroscopy. Carbohydr. Polym..

[65] Campos-Vallette M.M., Chandía N.P., Clavijo E., Leal D., Matsuhiro B., Osorio-Román I.O., Torres S. (2010). Characterization of sodium alginate and its block fractions by surface-enhanced Raman spectroscopy. J. Raman Spectrosc..

[66] Ramos P.E., Silva P., Alario M.M., Pastrana L.M., Teixeira J.A., Cerqueira M.A., Vicente A.A. (2018). Effect of alginate molecular weight and M/G ratio in beads properties foreseeing the protection of probiotics. Food Hydrocoll..

[67] Montanucci P., Terenzi S., Santi C., Pennoni I., Bini V., Pescara T., Basta G., Calafiore R. (2015). Insights in Behavior of Variably Formulated Alginate-Based Microcapsules for Cell Transplantation. BioMed Res. Int..

[68] Lee K.Y., Mooney D.J. (2012). Alginate: Properties and biomedical applications. Prog. Polym. Sci..

[69] Wen H., Xiao W., Biswas S., Cong Z.-Q., Liu X.-M., Lam K.S., Liao Y.-H., Deng W. (2019). Alginate Hydrogel Modified with a Ligand Interacting with α3β1 Integrin Receptor Promotes the Differentiation of 3D Neural Spheroids toward Oligodendrocytes in Vitro. ACS Appl. Mater. Interfaces.

[70] Mitry R.R., Jitraruch S., Iansante V., Dhawan A. (2017). Alginate Encapsulation of Human Hepatocytes and Assessment of Microbeads. Methods Mol. Biol..

[71] Zhao S., Agarwal P., Rao W., Huang H., Zhang R., Liu Z., Yu J., Weisleder N., Zhang W., He X. (2014). Coaxial electrospray of liquid core-hydrogel shell microcapsules for encapsulation and miniaturized 3D culture of pluripotent stem cells. Integr. Biol..

[72] Tarusin D., Mazur S., Volkova N., Yu P., Zaikov V., Petrenko A. (2016). Encapsulation of mesenchymal stromal cells in alginate microspheres. Biotechnol. Acta.

[73] Gryshkov O., Hofmann N., Lauterboeck L., Pogozhykh D., Mueller T., Glasmacher B. (2015). Multipotent stromal cells derived from common marmoset Callithrix jacchus within alginate 3D environment: Effect of cryopreservation procedures. Cryobiology.

[74] Mutsenko V., Tarusin D., Sydykov B., Zaragotas D., Simaioforidou A., Rozanski C., Gryshkov O., Wolkers W.F., Braslavsky I., Anastassopoulos E. (2018). Study of natural ice-nucleating agents for cryopreservation of 3D tissue-engineered scaffolds. Cryobiology.

[75] Zaragotas D., Liolios N.T., Anastassopoulos E. (2016). Supercooling, ice nucleation and crystal growth: A systematic study in plant samples. Cryobiology.

[76] Zheng G., Liu X., Wang X., Chen L., Xie H., Wang F., Zheng H., Yu W., Ma X. (2014). Improving stability and biocompatibility of alginate/chitosan microcapsule by fabricating bi-functional membrane. Macromol. Biosci..

[77] Bhattacharya S., Bozkurt Y. (2018). Cryopretectants and Their Usage in Cryopreservation Process. Cryopreservation Biotechnology in Biomedical and Biological Sciences.

[78] Solanki P.K., Bischof J.C., Rabin Y. (2017). Thermo-mechanical stress analysis of cryopreservation in cryobags and the potential benefit of nanowarming. Cryobiology.

[79] Eisenberg D.P., Bischof J.C., Rabin Y. (2016). Thermomechanical Stress in Cryopreservation Via Vitrification With Nanoparticle Heating as a Stress-Moderating Effect. J. Biomech. Eng..

[80] Tomás R.M.F., Bailey T.L., Hasan M., Gibson M.I. (2019). Extracellular Antifreeze Protein Significantly Enhances the Cryopreservation of Cell Monolayers. Biomacromolecules.

[81] Lauterboeck L., Hofmann N., Mueller T., Glasmacher B. (2015). Active control of the nucleation temperature enhances freezing survival of multipotent mesenchymal stromal cells. Cryobiology.

[82] Morris G.J., Acton E. (2013). Controlled ice nucleation in cryopreservation—A review. Cryobiology.

[83] Hunt C.J. (2019). Technical Considerations in the Freezing, Low-Temperature Storage and Thawing of Stem Cells for Cellular Therapies. Transfus. Med. Hemother..

[84] Chiu-Lam A., Staples E., Pepine C.J., Rinaldi C. (2021). Perfusion, cryopreservation, and nanowarming of whole hearts using colloidally stable magnetic cryopreservation agent solutions. Sci. Adv..

[85] Pogozhykh O., Pogozhykh D., Neehus A.-L., Hoffmann A., Blasczyk R., Müller T. (2015). Molecular and cellular characteristics of human and non-human primate multipotent stromal cells from the amnion and bone marrow during long term culture. Stem Cell Res. Ther..

[86] Ishikawa D., Diekmann U., Fiedler J., Just A., Thum T., Lenzen S., Naujok O. (2017). miRNome Profiling of Purified Endoderm and Mesoderm Differentiated from hESCs Reveals Functions of miR-483-3p and miR-1263 for Cell-Fate Decisions. Stem Cell Rep..

[87] Ahearne M., Yang Y., El Haj A.J., Then K.Y., Liu K.-K. (2005). Characterizing the viscoelastic properties of thin hydrogel-based constructs for tissue engineering applications. J. R. Soc. Interface.

[88] Tkalec G., Knez Ž., Novak Z. (2015). Formation of polysaccharide aerogels in ethanol. RSC Adv..

[89] Kilbride P., Morris G.J. (2017). Viscosities encountered during the cryopreservation of dimethyl sulphoxide systems. Cryobiology.

[90] Ehrlich L.E., Feig J.S.G., Schiffres S.N., Malen J.A., Rabin Y. (2015). Large Thermal Conductivity Differences between the Crystalline and Vitrified States of DMSO with Applications to Cryopreservation. PLoS ONE.

[91] López Molina J.A., Rivera M.J., Berjano E. (2014). Fourier, hyperbolic and relativistic heat transfer equations: A comparative analytical study. Proc. R. Soc. A.

