# Supplementary material for: Coaxial Alginate Hydrogels: From Self-Assembled 3D Cellular Constructs to Long-Term Storage
Source: Int J Mol Sci. 2021 Mar 18;22(6):3096. doi: 10.3390/ijms22063096 (PMC8003018; doi:10.3390/ijms22063096)
Supplement: Supplementary file 1 [file ijms-22-03096-s001.pdf]

International Journal of
Molecular Sciences

MDPI

Article

Coaxial Alginate Hydrogels: From Self-Assembled 3D Cellular Constructs to Long-Term Storage

Oleksandr Gryshkov [1,2,*], Vitalii Mutsenko [1,2], Dmytro Tarusin [3], Diaa Khayyat [1,2], Ortwin Naujok [4], Ekaterina Riabchenko [5], Yuliia Nemirovska [3], Arseny Danilov [5], Alexander Y. Petrenko [3] and Birgit Glasmacher [1,2]

Supplementary information

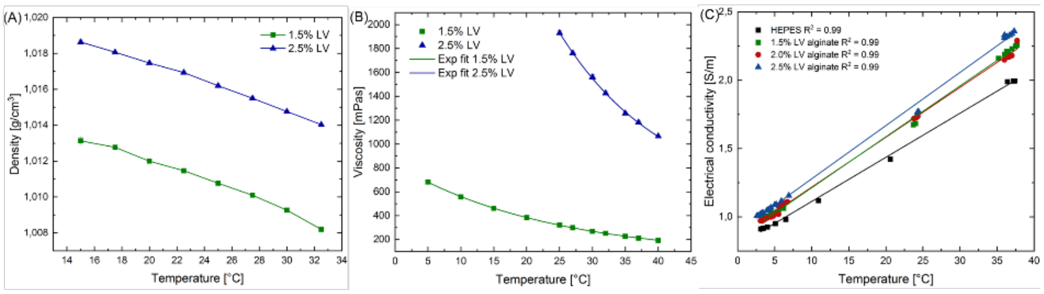

Figure S1. Effect of the alginate concentration and temperature on the density (**A**), viscosity (**B**) and electrical conductivity (**C**) of the LV alginate solution. Viscosity and conductivity curves were fit using an exponential and linear function, respectively.

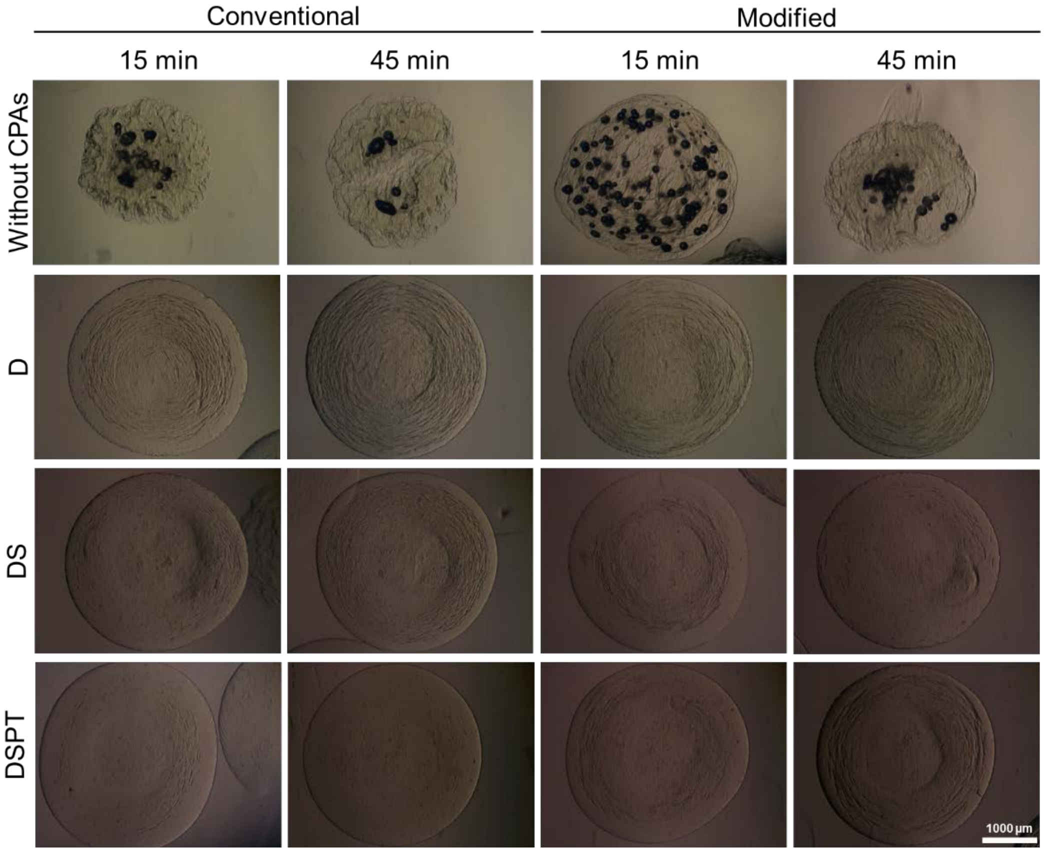

Figure S2. Bright-field images of core-shell capsules after cryopreservation. Samples were cryopreserved in the absence of CPAs (without CPAs), using 10% DMSO (D), 10% DMSO with 0.3 M sucrose without (DS) and with an additional pretreatment step with 0.1 M sucrose 24 h before freezing (DSPT) using the conventional and modified cryopreservation approaches. Time specification 15/45 min indicates CPA loading time before freezing. Scale bars are 1000 µm.

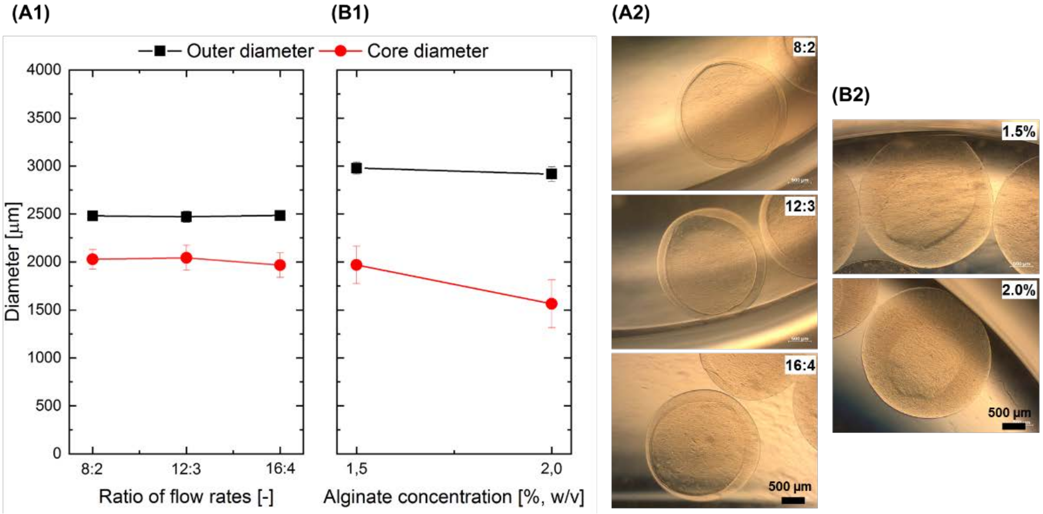

Figure S3. Effect of the absolute value of flow rates of the inner fluid and alginate (**A**, efficiency of scaling up) and alginate concentration (**B**) on the size of the alginate core-shell capsules. The capsules were made of 1.5% LV alginate using the following parameters: the distance between the inner and outer nozzle of 0.5 mm, spraying distance 7,5 cm, applied voltage 12,5 kV (**A**) and 10,0 kV (**B**), flow rate of the alginate inner fluid 14:2 (**B**). The effect of the increase in flow rates while keeping their ratio constant (**A1**, **A2**) as well as the effect of the alginate concentration increase (**B1**, **B2**) on the characteristics of the core-shell capsules are visible. Scale bars are 500 µm.

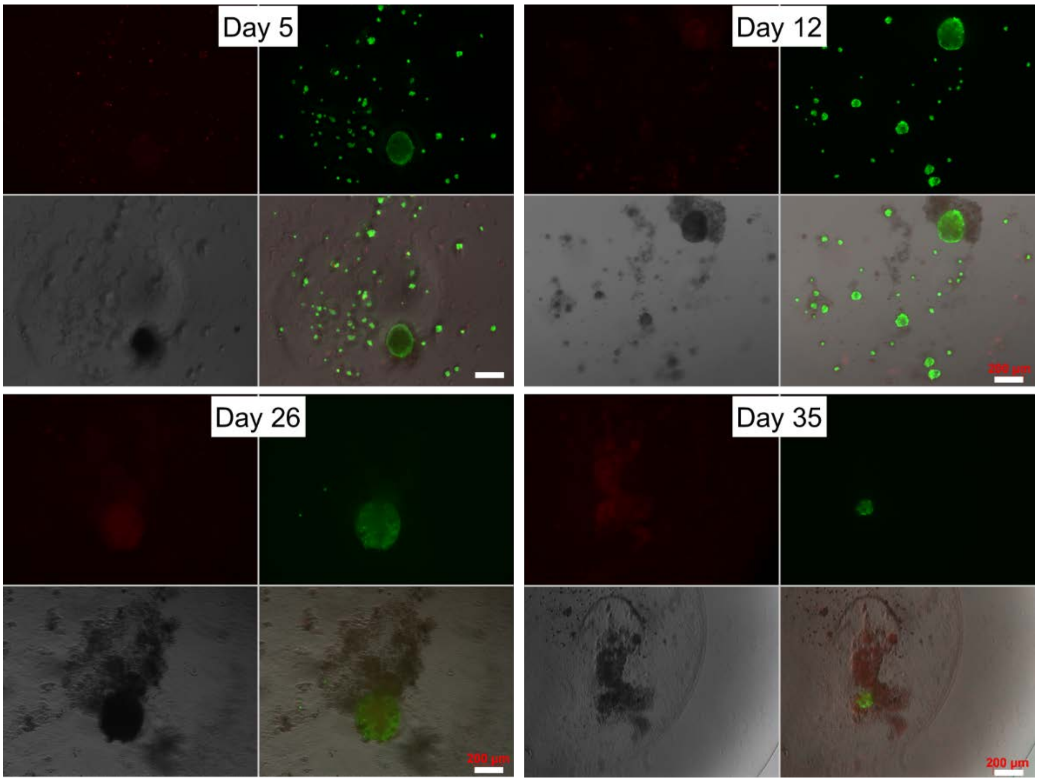

Figure S4. Representative fluorescent images of *cja*MSCs (35 days in culture) within the core-shell capsules produced from MV alginate. Green fluorescence shows live cells, whereas red fluorescence – dead cells. Scale bars are 200 µm.

Modelling of the temperature and mechanical stress distribution

A mathematical model of the heat transfer was built to evaluate the temperature and mechanical stress distribution during freezing in a model core-shell capsule using COMSOL Multiphysics. For the model core-shell capsule, the axis-symmetric radial geometry was chosen. A capsule was pre-

sented as a union of two concentric spheres, in which the internal sphere with the radius R2 represents an inner core of a capsule filled with the cryoprotectant 10% DMSO whereas an outer layer with the major radius R1 – outer diameter of a capsule (the difference R1-R2 is equal to the alginate membrane thickness). The geometry of the heat transfer model is presented in Figure S5. "Heat transfer in solids" and "Solid mechanics" modules were used to obtain heat and stress maps. The following physical properties of calcium alginate hydrogel and a CPA solution (either taken from the sources [78,87–91] or in part experimentally determined) were used in the heat transfer modelling: density, viscosity, thermal conductivity, specific heat capacity, Young's modulus and coefficients of thermal expansion. Modelling was conducted for the slow freezing scenario from 4 °C to -196 °C at a cooling rate of 1 K/min. For thawing, the boundary condition for the temperature was set to 37 °C at the outer membrane interface.

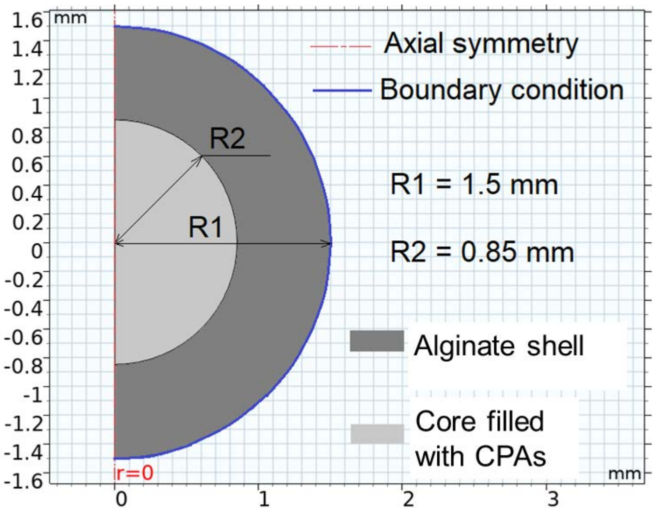

Figure S5. Geometry of heat transfer model

Heat transfer in a model object presented in Figure S5 can be described by the inhomogeneous Fourier heat equation [71]:

$$c \times \varrho \times (\partial T(r,z,t)/\partial t) = k \times \Delta T(r,z,t) + f(r,z,t), \quad (1)$$

where c is the specific heat of a material, ϱ is the density, k is the thermal conductivity, f(r,z,t) is the heat source.

It should be noted that the core-shell capsule was modelled as a solid taking into account the viscoelastic properties of the material. Therefore, displacements in geometry shown in Figure S5 may be described by the following equation:

$$\varrho \times (\partial^2 \mathbf{u}(r,z,t)/\partial t^2) = \mathrm{div}S + \mathbf{F}_V, \quad (2)$$

where **u** is a displacement vector, S is a second-order tensor field, $\mathbf{F}_V$ is the volumetric force.
